# Untargeted metabolomic profiling of childhood asthma: An exploratory analysis of anthropogenic chemicals and the serum metabolome

**DOI:** 10.1097/EE9.0000000000000480

**Published:** 2026-05-08

**Authors:** Max J. Oosterwegel, Dorina Ibi, Ulrike Gehring, Gerard H. Koppelman, Judith M. Vonk, Jolanda M.A. Boer, Jelle Vlaanderen, Kathryn Dunn, Brismar Pinto-Pacheco, Douglas I. Walker, Roel Vermeulen

**Affiliations:** a Institute for Risk Assessment Sciences, Utrecht University, Utrecht, The Netherlands; b Department of Pediatric Pulmonology and Pediatric Allergology, GRIAC Research Institute, University of Groningen, University Medical Center Groningen, Beatrix Children’s Hospital, Groningen, The Netherlands; c Department of Epidemiology and GRIAC Research Institute, University of Groningen, University Medical Center Groningen, Groningen, The Netherlands; d Centre for Prevention, Lifestyle and Health National Institute for Public Health and the Environment, Bilthoven, The Netherlands; e Department of Environmental Medicine and Climate Science, Icahn School of Medicine at Mount Sinai, New York, NY; f Gangarosa Department of Environmental Health, Rollins School of Public Health, Emory University, Atlanta, Georgia; g Julius Center for Health Sciences and Primary Care, University Medical Center Utrecht, Utrecht, The Netherlands

**Keywords:** metabolomics, exposome, asthma, PFAS, phthalates, pesticides, phenols, children

## Abstract

**Background:**

Asthma is the most common chronic disease in children, yet its causes, environmental links, and underlying mechanisms are still not well understood despite extensive research.

**Methods:**

We examined the cross-sectional relationship between asthma and metabolic features in 628 serum samples (165 cases, 463 controls) from children aged 8, 12, and 16 years, in the Prevention and Incidence of Asthma and Mite Allergy birth cohort. Metabolic features were assessed using liquid chromatography with high-resolution mass spectrometry. In a single-feature-at-a-time approach, asthma status (i.e., current asthma) was regressed against the measured intensity of each of the features; this approach alone was further extended to age-stratified analyses. Biological pathways were explored using Mummichog. In addition, we assessed the association of exogenous mixtures exhibiting substantial intercorrelations (i.e., for polyfluoroalkyl substances [PFAS] only) with asthma.

**Results:**

Liquid chromatography with high-resolution mass spectrometry detected 55,444 metabolic features, including 38 identified exogenous compounds (15 PFAS, 14 pesticides, 4 phenols, 4 phthalates, and 1 other compound) and 460 identified endogenous metabolites. Overall, we observed limited evidence of robust associations between individual environmental compounds and childhood asthma. Some age-specific signals were observed, including a positive association for monocyclohexyl phthalate and a negative association for monoethyl phosphate in age-stratified analyses, although these findings did not consistently meet multiple-testing thresholds. PFAS as a mixture was not associated with asthma (*P* = 0.67, odds ratio = 1.00). Pathway analyses indicated potential involvement of the tyrosine metabolism pathway in relation to asthma and several environmental compounds.

**Conclusion:**

In this exploratory metabolomics analysis, we found limited evidence for strong associations between measured environmental compounds and childhood asthma. Nevertheless, several age-specific signals and pathway-level patterns, particularly involving tyrosine metabolism, were observed and may help guide future hypothesis-driven studies.

**What this study adds**

This work offers several novel contributions. First, it utilizes an unbiased liquid chromatography with high-resolution mass spectrometry approach to simultaneously capture thousands of metabolic features, mitigating selective reporting and enabling a broader assessment of metabolic processes potentially relevant to asthma etiology. This approach also allows the simultaneous evaluation of a wide range of anthropogenic chemicals. Second, by highlighting metabolic pathways that may be related to asthma and environmental exposures in exploratory analyses, the study identifies metabolic pathways that may inform future investigations into mechanisms underlying asthma. Together, these contributions help address gaps in the understanding of asthma’s environmental determinants and illustrate the value of untargeted metabolomic profiling for investigating complex relationships between multiple environmental exposures and the human metabolome.

## Introduction

Asthma is the most prevalent chronic disease in children, characterized by chronic inflammation of the airways.^1^ The prevalence in children is estimated to be approximately 9% worldwide, but differs substantially between regions, ranging from less than 1% (Lucknow, India) to as high as 29% (Costa Rica), with the Netherlands reporting a prevalence of approximately 6%.^2–4^ Asthma prevalence has been stable over the last decades after a substantial increase over the second half of the 20th century.^3^ Despite large research efforts, much remains unknown about the causes of asthma in children and the role of environmental exposures in particular.^5^ Previous studies have reported a possible link between asthma and exposures to compounds such as pesticides,^6^ phthalates,^7^ and per- and polyfluoroalkyl substances (PFAS).^8^ However, the evidence remains inconclusive, often characterized by conflicting findings and differences among the populations studied, ranging from occupational cohorts to children.^9,10^

Comprehensive profiling of serum samples using nontargeted liquid chromatography with high-resolution mass spectrometry (LC-HRMS) provides an opportunity for simultaneous assessment of hundreds of endogenous and exogenous compounds and their relationship with asthma, thereby reducing selective reporting. Moreover, the metabolic information captured by the LC-HRMS features can provide insight into biological processes related to asthma.

In this research, we used LC-HRMS data from 628 serum samples from a nested case–control study within the Prevention and Incidence of Asthma and Mite Allergy birth cohort^11^ to examine the cross-sectional relationships between asthma status and 55,444 metabolic features at ages 8, 12, and 16 years. These features include 38 exogenous compounds (15 PFAS, 14 pesticides, four phenols, four phthalate compounds, and one other)—originating from anthropogenic sources—and 460 endogenous metabolites, identified using authentic reference standards (Schymanski level 1). Given the large number of measured features, this analysis was exploratory and aimed to identify potential metabolic signals and pathways that may relate to asthma and environmental exposures. To assess potential age-related differences, we examined these relationships separately for each age group. We additionally evaluated the relationship between asthma and a mixture of identified PFAS and conducted pathway analyses to explore metabolic pathways linked to both asthma and the detected exogenous compounds.

## Methods

### Study population

The Prevention and Incidence of Asthma and Mite Allergy birth cohort study has been described in detail before.^11^ In brief, participants were recruited from the general population through antenatal clinics in three different regions of the Netherlands (north, center, and west) in 1996–1997, resulting in 3963 children at baseline. Since baseline, parents and the participants themselves, from age 11 onwards, have been asked to complete questionnaires (yearly until age 8 and then approximately every 3 years). At ages 8, 12, and 16, serum samples were collected during clinical examinations.

### Nested case–control study

This nested case–control study was performed among children of Dutch parents with serum available at ages 8, 12, and/or 16 years. Asthma cases at ages 8, 11, and 16 years were defined as fulfilling at least two of the following three criteria as reported in the questionnaires at the respective follow-ups: wheezing in the past 12 months, doctor-diagnosed asthma ever, and asthma medication in the past 12 months.^12^ Controls were defined as being nonasthmatic during the entire follow-up until age 16. All available serum samples from cases were selected for this study. Control serum samples (n = 463) were matched on sex and follow-up round to the case serum samples (n = 165). Cases with repeated samples were matched to the same control at the various time points as much as possible. Serum samples were collected from 2005 to 2014.

### Metabolomic analysis

#### Sample processing

After blood collection, the sample stayed at room temperature for 30 minutes to allow clotting. Then the sample was centrifuged to separate serum from cells. Serum was then transferred to Sarstedt freezer tubes and immediately stored at −80°C until the moment of transfer to the lab, and subsequent analysis. For the analysis, samples were prepared according to an established protocol, and untargeted serum metabolomic profiles were assessed using LC-HRMS.^13^ More specifically, before analysis, serum samples were thawed at 4°C, and 30 μL serum was extracted by adding 90 μL acetonitrile containing 13C-labeled internal standards. Treated samples were vortexed for 2 minutes, equilibrated at 4*°*C for 30 minutes, and then centrifuged for 45 minutes at 3,220 × g at 4°C. Two aliquots of supernatant (30 μL) were then transferred to 96-well plates containing 60 μL water (C18) or 60 μL 1:1 acetonitrile/water (hydrophilic interaction liquid chromatography). These were vortexed for 2 minutes and placed in a refrigerated autosampler until analysis. Samples were then analyzed in triplicate, operated with hydrophilic interaction liquid chromatography and C18 hydrophobic reversed-phase chromatography in positive and negative electrospray ionization modes, resulting in four total analyses (Thermo Scientific Vanquish Duo LC system & Q Exactive HF-X Orbitrap MS system). To maintain data quality during the metabolomic experiment, QA/QC samples consisting of replicate analyses of two pooled serum samples were placed at the beginning, end, and every 20 study samples within each 96-well plate; NIST SRM 1950 was analyzed at the beginning and end of each study run; and method blanks were analyzed at the beginning and end of each batch. QA/QC analysis was completed daily using an automated workflow combining TraceFinder with automatic report generation, and was verified by at least two different individuals.

After quality control, metabolite features were extracted from the data using apLCMS with modifications by XMSanalyzer. This resulted in ion intensity information on 63,129 features identifiable by their mass and retention time. After excluding the features that were detected in less than 30% (= approximate proportion of cases in study) of the samples, 55,444 features remained. Confirmed compounds (Schymanski level 1) were identified by comparing detected *m/z* and retention time to a database of 1200 standards analyzed using the same method parameters that included a wide range of environmental and endogenous compounds. Metabolite identifications were determined by matching *m/z* and retention time with a tolerance of 5 ppm and 15 seconds, respectively.

### Statistical analysis

#### Imputation of features

After the sample processing, nondetectable values of a feature were imputed using a procedure similar to Lubin et al^14^ This approach assumes that nondetectable values are due to left-censoring at the instrument’s detection limit and not due to interfering analytes. Specifically, we fitted parametric models to the left-censored natural log-transformed features (one feature at a time) with asthma status, sex, a residual term for age, follow-up round (and two-way interactions of follow-up round and the other fixed effect predictors in the model to allow for different effects per follow-up round), a random intercept term for batch and a random intercept for subject as predictors. The follow-up round variable and its interaction terms with the other variables were regularized with a Gaussian prior with a mean of zero and a standard deviation of three to slightly regularize large coefficients for these terms when data in a follow-up round were sparse. We defined the limit of detection as the minimum observed (log-transformed) intensity value of a batch. Subsequently, we imputed the nondetectable values by taking 70 random draws (i.e., 100 - minimum detection threshold of 30%) below the batch-adjusted detection limit from the posterior of this fitted model. This resulted in 70 imputed datasets for each feature with nondetectable values.

#### Batch-correction intensity values

After batch adjustment using ComBat,^15^ there were still batch effects present in features with nondetectable values (n = 52,456 [94.6%]). Therefore, each feature with nondetectable values was adjusted for possible batch effects by subtracting the random batch intercept from the observed (or imputed) value. Imputation models were fitted using the brms package, which provides an interface to fit Bayesian models using the full Bayesian inference tool Stan.^16,17^

#### Associations of single features with asthma

We used Firth’s logistic regression to estimate the associations between asthma status of the sample and the intensity of each feature (single-feature-at-a-time). Each of the models had asthma as outcome, the natural log-transformed feature intensity as predictor of interest, indicators of the matching variables (sex and follow-up round), and the residual age (actual age minus the rounded age [no decimals] used for matching) as covariates. Stratified analyses by age (8, 12, and 16) only included the natural log-transformed feature intensity and sex as predictors.

Results of the logistic regressions for each of the features across the different imputed datasets were combined using Rubin’s rules.^18^ The false discovery rate (FDR) was controlled separately per analysis (main and stratified) and per compound group (exogenous and endogenous) at 10% using the Benjamini–Hochberg procedure. The results from the identified features could be interpreted directly, while the results for the unidentified features were only used in the pathway analysis (see “Pathway analysis”).

#### Associations of exogenous mixtures with asthma

For exogenous compound classes whose members showed substantial intercorrelations (ρ> ≈0.3) upon visual inspection of a correlation plot, we used g-computation to estimate a joint association of the components of the mixture and asthma.^19,20,21^ In other words, we fitted a simple logistic regression model with all the components of the mixture and estimated the odds of asthma associated with a one unit increase of all the components of the mixture by calculating the mixture’s marginal effect. With a simple logistic regression, this marginal effect is equal to the sum of the regression coefficients for the components in the mixture. Further details on our implementation can be found in Supplementary Methods I, https://links.lww.com/EE/A427.

#### Pathway analysis

We performed a pathway analysis on our results of the single feature at a time analysis of all features (both identified and unidentified) and asthma to extract meaning from the great number of unidentified features. This was done with the mummichog tool (via MetaboAnalystR). Mummichog predicts functional activity directly from the feature table without the certain identification of those features.^22^ The *P*-value of a feature’s relationship with asthma status, the feature’s m/z value and retention time, and the electrospray ionization mode used to produce the feature were used as input. Parameters of mummichog were set to 1000 permutations, a mass tolerance of 5 ppm, and mixed analytical mode. Features significant at an FDR of 10% or—if this yielded less than 1% of the features—the features in the 1% percentile of *P*-values were used to evaluate pathway enrichment.

In a meet-in-the-middle approach, we also assessed the relationship between the identified exogenous compounds and the endogenous features (both identified and unidentified), and used these results for a pathway analysis per identified exogenous compound. See Supplementary Methods II, https://links.lww.com/EE/A427 for details.

The results of each pathway analysis were adjusted for multiple testing against all the pathways using the Benjamini–Hochberg procedure at an FDR of 10%. This was done separately for the asthma pathways and separately for each of the exogenous compound pathways.

#### Temporal variability of exogenous compound measurements

To assess how variable our measurements of the identified exogenous compounds were over a 4-year period, we calculated intraclass correlation coefficients (ICCs) for each of the identified exogenous compounds using data from participants with repeat measurements at the follow-ups at ages 12 and 16 years (= age groups with the greatest numbers of repeats, see Figure S1, https://links.lww.com/EE/A427). Briefly, we fitted the same type of model as described in the “Imputation of features” section to each identified exogenous compound and calculated an ICC by dividing the random subject intercept variance by the random subject intercept variance plus the residual error variance, thereby considering the batch variance as a nuisance term. Further details can be found in Supplementary Methods III, https://links.lww.com/EE/A427.

## Results

### Study population

In total, 628 serum samples were included in this study. These samples were obtained from a total of 523 subjects; 99 subjects provided serum samples for two follow-ups, three provided samples for all three follow-ups, and 421 provided a single serum sample (details in Venn diagram of Figure S1, https://links.lww.com/EE/A427). Characteristics of the study sample are shown in Table 1. In brief, most serum samples were from boys (56%). In total, 165 serum samples were from cases and 463 from controls. From the 628 serum samples, 118 samples were collected at the follow-up at age 8 years, 200 at age 12 years, and 310 at age 16 years. Detailed cohort information (e.g., wheezing phenotypes) is available in Wijga et al.^11^

**Table 1.** Characteristics of the study sample. Median [IQR]; n (%).

	Total		Per follow-up round					
	8, 12, and 16 years		8 years		12 years		16 years	
	Controls (n = 463)	Cases (n = 165)	Controls (n = 89)	Cases (n = 29)	Controls (n = 120)	Cases (n = 80)	Controls (n = 254)	Cases (n = 56)
Age subject (years)	16.09 (3.93)	12.64 (3.95)	8.11 (0.51)	8.61 (0.74)	12.61 (0.62)	12.54 (0.45)	16.33 (0.27)	16.31 (0.17)
Sex subject								
Boy	252 (54%)	100 (61%)	44 (49%)	17 (59%)	67 (56%)	51 (64%)	141 (56%)	32 (57%)
Girl	211 (46%)	65 (39%)	45 (51%)	12 (41%)	53 (44%)	29 (36%)	113 (44%)	24 (43%)

### Assessment of blood metabolome

From the 55,444 features, 38 exogenous metabolites (metabolites of a compound that the human body cannot produce) could be confidently identified, matching against our database of 1200 standards. These metabolites belonged to four chemical classes (15 PFAS, 14 pesticides, 4 phenols, 4 phthalates, and 1 other compound).

 687 features could additionally be identified. These features referred to 460 unique endogenous metabolites. Tables S1a and S1b, https://links.lww.com/EE/A427 contain full lists of identified compounds and the proportion of samples in which these compounds were detected, while Figure S2, https://links.lww.com/EE/A427 shows the distribution of the proportion of nondetects across all features.

### Evaluation of single metabolic features in relation to asthma

**Figure 1.**
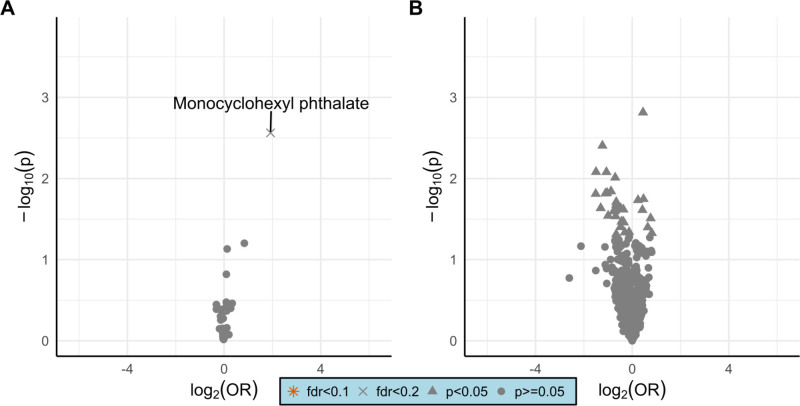
Volcano plot of the associations between asthma and the identified compounds: (A) exogenous compounds, (B) endogenous compounds. FDR indicates false discovery rate as controlled by the Benjamini–Hochberg procedure; OR, odds ratio per one natural log unit change in compound intensity.

Monocyclohexyl phthalate (MCHP) showed the strongest signal among the identified exogenous compounds in relation to asthma (odds ratio [OR] = 3.79, unadjusted 95% confidence interval [CI] = 1.59, 9.06, *P-value* = 0.003), although this association was not statistically significant after controlling the FDR at 10% (FDR-adjusted *P-value* = 0.104). Various endogenous compounds were associated with asthma status, but none were statistically significant after controlling the FDR (smallest FDR adjusted *P-value* = 0.847). The volcano plots in Figure 1 show the full distribution of *P*-values and OR for this analysis.

#### Age-stratified analysis

In the stratified analyses by age monoethyl phosphate was statistically significantly associated with asthma at age 8 (FDR adjusted *P-value* = 0.046, OR = 0.10, unadjusted 95% CI = 0.03, 0.4), but not at ages 12 and 16 (12 years: FDR adjusted *P-value* = 0.496, OR = 1.89, unadjusted 95% CI = 0.91, 3.96; 16 years: FDR adjusted *P-value* = 0.998, OR = 0.69, unadjusted 95% CI = 0.23, 2.09). At age 16, MCHP was statistically significant (FDR adjusted *P-value* = 0.053, OR = 12.68, unadjusted 95% CI = 2.67, 60.16), but not at age 8 and 12 (8 years: FDR adjusted *P*-value = 0.672, OR = 0.46, unadjusted 95% CI= 0.10, 2.23; 12 years: FDR adjusted *P* value = 0.102, OR = 8.11, unadjusted 95% CI = 1.72, 38.29).

Various endogenous compounds were associated with asthma at different ages, but none were statistically significant after controlling the FDR (smallest *P*-values after FDR adjustment were 0.190, 0.522, and 0.937 at ages 8, 12, and 16 years, respectively). Volcano plots of the stratified analysis can be found in Figure S3, https://links.lww.com/EE/A427. All results of the exogenous compound are in Table S2, https://links.lww.com/EE/A427.

#### Associations of exogenous mixtures with asthma

Correlations between the exogenous compounds were generally low within chemical classes (correlation between 0 and 0.2), except within the group of PFAS compounds, which, as expected, showed substantial correlation between the compounds in the class (numerous correlations > 0.3) (Figure S4, https://links.lww.com/EE/A427).

The joint association between this mixture of the PFAS compounds and asthma status was not statistically significant (*P* = 0.67, OR = 1.00), in line with the absence of nominally significant associations for any individual compound (smallest *P* = 0.07 for perfluorobutanesulfonic acid).

### Pathway analysis related to asthma and environmental compounds

Based on the associations we observed between the non-exogenous metabolic features and asthma, the tyrosine metabolism pathway emerged in pathway enrichment analysis in relation to asthma status at an FDR of 10% (Table S3f, https://links.lww.com/EE/A427).

Based on the associations we observed between 15 pesticides and the metabolic features, 35 biological pathways were putatively enriched at an FDR of 10%. The pathways associated with the most pesticide compounds were arachidonic acid metabolism (n = 6 of 14 compounds), linoleate metabolism (n = 6/14), and prostaglandin formation from the arachidonate pathway (n = 5/14) (Table S3a, https://links.lww.com/EE/A427). The measured PFAS compounds were associated with 37 biological pathways at an FDR of 10%. Most common were linoleate metabolism (n = 9/15), arachidonic acid metabolism (n = 8/15), and butanoate metabolism (n = 8/15) (Table S3b, https://links.lww.com/EE/A427). The measured phenol compounds were related to 13 biological pathways, but no single pathway was related to multiple phenol compounds (Table S3c, https://links.lww.com/EE/A427). The measured phthalate compounds were associated with 14 biological pathways at an FDR of 10%. Most common were androgen and estrogen biosynthesis and metabolism (n = 2/4), arginine and proline metabolism (n = 2/4), and C21-steroid hormone biosynthesis and metabolism (n = 2/4) (Table S3d, https://links.lww.com/EE/A427). Monoethyl phosphate was associated with 9 biological pathways (Table S3e, https://links.lww.com/EE/A427).

The exogenous compounds isoprocarb, acetamiprid, 2-aminohexafluoropropan-2-ol, and mono-2-heptyl phthalate were also associated with the tyrosine pathway. The full results of the pathway analyses can be found in Table S3, https://links.lww.com/EE/A427.

### Temporal variability of exogenous compound measurements

The median ICC over 4 years (age 12 and 16) for the exogenous compounds was 0.09 (IQR, 0.17), indicating substantial variation in exposure between the two time points. The ICC was lowest for compounds from the pesticide and PFAS classes (median ICC = 0.07) and highest for compounds from the phenol class (median ICC = 0.23). Overall, the ICC estimates varied considerably within most chemical classes. The ICC estimate was highest for the aldicarb-sulfone compound with 0.53. Detailed results can be found in Figure S5, https://links.lww.com/EE/A427.

## Discussion

In this cross-sectional analysis of serum metabolomic profiles and environmental compounds in children aged 8, 12, and 16 years, we found overall limited evidence for strong associations between individual exogenous compounds and asthma. Some age-specific signals emerged in stratified analyses, including a positive association for MCHP at age 16 and a negative association for monoethyl phosphate at age 8. However, these findings were not consistently significant after correction for multiple testing and should therefore be interpreted cautiously. The tyrosine metabolism pathway emerged in pathway enrichment analysis in relation to asthma and several environmental compounds, suggesting a potential metabolic context that may warrant further investigation. Our findings, therefore, primarily provide hypothesis-generating signals rather than definitive evidence of associations.

The observed association between asthma and the tyrosine metabolism pathway is consistent with findings of a previous review that noted evidence of an altered tyrosine pathway in asthmatic children.^23^ Tyrosine metabolism involves the breakdown and conversion of the tyrosine amino acid into melanin pigments or into neurotransmitters such as dopamine, adrenaline, and noradrenaline. The pathway also plays an important role in the synthesis of thyroid hormones and the generation of catecholamines. A previous study found subjects with autoimmune thyroid disorders had higher odds of having asthma than controls, but the exact role of thyroid hormones in asthma pathobiology is unclear.^24,25^ Catecholamines are produced by the body in response to stress and also play a role in the respiratory rate and bronchoconstriction.^26,27^ Interestingly, tyrosine metabolism has also been linked to air pollution exposure and corticosteroid resistance in asthmatic children.^28,29^ None of the exogenous compounds associated with tyrosine metabolism in our study (i.e., isoprocarb, acetamiprid, mono-2-heptyl phthalate, 2-aminohexafluoropropan-2-ol) have been previously identified in research on childhood asthma, potentially pointing to novel risk factors for asthma.

Evidence linking phthalate exposure to asthma remains inconsistent. Experimental studies have suggested potential roles of phthalates in allergic sensitization, and epidemiological studies have reported associations between polyvinyl chloride flooring and childhood asthma.^30,31^ In contrast, biomonitoring studies measuring phthalates in biospecimens have generally shown less consistent associations.^32,33^ In our analysis, we found overall limited evidence for associations between phthalate-related compounds and asthma. However, MCHP showed a positive association with asthma in age-stratified analyses, although this finding did not consistently remain significant after correction for multiple testing and should therefore be interpreted as exploratory. We also observed a negative association between monoethyl phosphate and asthma at age 8. To our knowledge, the relationship between this compound and respiratory health has not been previously examined.

Our findings for PFAS, pesticides, and phenols seem consistent with current evidence. For example, Van Holst et al^34^ noted that convincing evidence for an association between asthma and PFAS levels in children is scarce. Similarly, epidemiological studies examining pesticides and phenolic compounds have generally reported inconsistent evidence.^8,35^ However, it is worth mentioning that studying the etiology of asthma is difficult with its heterogeneous clinical phenotype and its periods of possible remission and relapse.^5^ This makes it harder to consider the absence of evidence as possible evidence of the absence of an effect.

Our analysis showed several biological mechanisms being related to the studied exogenous compounds. In total, 52 biological pathways were associated with these compounds. Most central to these perturbations were arachidonic acid metabolism (n = 17 compounds), linoleate metabolism (n = 17), butanoate metabolism (n = 15), beta-alanine metabolism (n = 11), leukotriene metabolism (n = 11), and the prostaglandin formation from the arachidonate pathway (n = 11). All but butanoate metabolism (related to fatty acid synthesis) and the beta-alanine metabolism pathway (related to diet) are related to inflammation. Inflammation is a hallmark of environmental diseases,^36^ and both inflammation and fatty acids play a central role in asthma.^37,38^ These findings suggest that the studied exogenous compounds may contribute to asthma by influencing these pathways and promoting (chronic) inflammation.

Although there were similarities between the different compounds and the biological associations, there were also notable differences. For example, the phthalates were associated with several hormonal pathways, including androgen and estrogen biosynthesis and metabolism, and C21-steroid hormone biosynthesis and metabolism. Interestingly, phthalates have been shown to disrupt endocrine function, increasing our confidence in the pathway analysis results.

Our study has several advantages. First, we simultaneously assessed the relationship of asthma with many exogenous and endogenous compounds and used objective quantitative measurements. This limits the selective reporting of results and the chances of self-report bias. Furthermore, we assessed the associations of compounds with asthma in a mixture that considers simultaneous exposure to those compounds, thereby possibly better reflecting real-world exposure scenarios. Additionally, our pathway analysis enabled us to extract information on possible biological activity from the many unidentified features.

There are, however, also limitations worth noting. Given the large number of tested metabolic features and the exploratory nature of untargeted metabolomics analyses, some observed signals may reflect chance findings and therefore require replication in independent cohorts. First, it is unclear what period of (internal) exposure a single measure in serum reflects. Previous LC-HRMS analyses have shown fair correlations between repeated measurements in serum samples of an individual collected over a period of multiple months, while our low ICC values over 4 years for the exogenous compounds suggest that our measurements are a relatively poor reflection of longer-term averages.^39^ Measurement error is unavoidable when using a single measure of exposures, and this will have diluted a possible signal of a long-term exposure effect in our analysis. Second, our assessment of the association between a mixture and asthma did not explore interactions between the compounds of the mixture or nonlinear relationships between the compounds and asthma because of concern with sparse data bias and overfitting. Nonetheless, we think that our assumption of a linear relationship is a reasonable starting point.^40^ Furthermore, in all our analyses of asthma, we treated the samples as independent, meaning that we did not consider a possible correlation among serum samples of the same individual. A common remedy, such as multilevel logistic regression, was not possible in our study because a case was always a case and a control was always a control, which meant that there was no variation in case status within a subject, causing complete separation in these multilevel models.^41^ A likely consequence of our approach is an underestimation of the standard errors in these analyses. Finally, unobserved confounding (e.g., diet and tyrosine metabolism) cannot be excluded.

In this exploratory metabolomics study, we observed limited evidence for strong associations between individual environmental compounds and childhood asthma. Although most associations were null after multiple-testing correction, several age-specific signals and pathway-level patterns, particularly involving tyrosine metabolism, were identified. These findings should be interpreted cautiously but may provide useful leads for future hypothesis-driven and longitudinal studies.

## Conflicts of interest statement

The authors declare that they have no conflicts of interest with regard to the content of this report.

## ACKNOWLEDGMENTS

*We greatly acknowledge all those who are responsible for data collection and management in the PIAMA study*.

## Supplementary Material
