# Supplementary material for: Untargeted metabolomic profiling of childhood asthma: An exploratory analysis of anthropogenic chemicals and the serum metabolome
Source: Environ Epidemiol. 2026 May 8;10(3):e480. doi: 10.1097/EE9.0000000000000480 (PMC13155524; doi:10.1097/EE9.0000000000000480)

Untargeted Metabolomic Profiling of Childhood Asthma: An Exploratory Analysis of Anthropogenic Chemicals and the Serum Metabolome – Supplementary Materials

Max J. Oosterwegel[1], Dorina Ibi[1], Ulrike Gehring[1], Gerard H. Koppelman[2], Judith M. Vonk[3], Jolanda M.A. Boer[4], Jelle Vlaanderen[1], Kathryn Dunn[5], Brismar Pinto-Pacheco[5], Douglas I. Walker[6], Roel Vermeulen[1,7*]

1 Institute for Risk Assessment Sciences, Utrecht University, Utrecht, 3584 CM, The Netherlands.

2 University of Groningen, University Medical Center Groningen, Beatrix Children's Hospital, Department of Pediatric Pulmonology and Pediatric Allergology and GRIAC Research Institute, Groningen, The Netherlands.

3 University of Groningen, University Medical Center Groningen, Department of Epidemiology and GRIAC Research Institute, Groningen, The Netherlands.

4 Centre for Prevention, Lifestyle and Health National Institute for Public Health and the Environment.

5 Department of Environmental Medicine and Climate Science, Icahn School of Medicine at Mount Sinai, New York, NY

6 Gangarosa Department of Environmental Health, Rollins School of Public Health, Emory University, Atlanta, GA, United States.

7 Julius Center for Health Sciences and Primary Care, University Medical Center Utrecht, Utrecht, 3508 GA, The Netherlands.

** Corresponding author. Email address: r.c.h.vermeulen@uu.nl*

Summary: 34 pages, 4 figures, 3 tables.

Supplementary Methods I: multiple features at a time

To provide an answer to the question on mixtures a separate imputation procedure was setup. Specifically, we imputed values below the limit of detection of left-censored features by using all the other (possibly also censored) features of the mixture and case status, wave of follow-up (as factor), sex, and the residual age (actual age minus the rounded age (no decimals) used for matching) as predictors in a multivariate imputation by chained equation procedure using the mice package.

This procedure starts with an initial 'placeholder' value for the left-censored observations and use these initial values to iteratively replace the placeholders using new fits of a tobit model. We used the tobit model as implemented in the leftcensnorm.mice function from qgcomp to draw imputations below the limit of detection of a feature. This function corresponds to the model of Lubin et al. (2004) without the bootstrap [14]. The limit of detection was defined as the smallest detected intensity value of a batch. 10 iterations were performed per imputed dataset and 70 imputed datasets were generated. Subsequently the following model was fitted to all imputed datasets using Firth's logistic regression model:

Asthma ~ 1 + wave + sex + age_residual + feature_1 + … + feature_k

where the features are all compounds of the PFAS class.

Lastly, using these fitted models a marginal joint association of the components of the feature and case status was calculated using the marginaleffects package.[41]

Supplementary Methods II: estimating relationship exogenous compounds and endogenous compound features

The challenge with assessing the relationship between two features is that both features can be censored, so called bivariate censoring. In those cases standard tobit models like we used in the imputation for the main analysis do not work out of the box. To overcome this we set up a multivariate imputation by chained equations procedure where we start with an initial 'placeholder' value for the left-censored observations and use these initial values to iteratively replace the placeholders using new fits of a tobit model. This tobit model included the other (possibly censored) feature, the wave of follow-up (as factor), sex and the residual age (actual age minus the rounded age (no decimals) used for matching) as predictors. We defined the limit of detection was defined as the smallest detected intensity value of a batch. This was done for 10 iterations for each censored feature. After 10 iterations the process was assumed to be converged and the final imputed dataset of a chemical and metabolic feature relationship was extracted. This procedure was performed using the mice package with the leftcensnorm.mice function from qgcomp.

After imputing values below the detection limit we fit a simple model of

log(M) ~ 1 + log(E) + wave + sex + age_residual.

Where M is an unidentified feature, and E is identified exogenous compound and its estimated parameter the coefficient of interest.

The described procedure was performed for the relationship of all identified exogenous compounds and unidentified features (so 38 * ~55k). FDR was controlled per exogenous compound using the Benjamini-Hochberg procedure.

Supplementary Methods III: intraclass correlation coefficient calculation (ICC)

To estimate an ICC value for each of the exogenous compounds we fitted the following model to data of subjects that provided a serum sample at both 12 and 16 years old:

$$\log(y_{ijk}) = \beta + u_j^{(2a)} + u_k^{(2b)} + \epsilon_{ijk}^{(1)} \quad (1)$$

for intensity measurements $i = 1, \ldots, n_{jk}$ and level-2 groups (subjects) $j = 1, \ldots, M_1$ and a level-2 group for the batch an observation was in $k = 1, \ldots, M_2$. We assumed the error term and level-2 random intercepts to be normally distributed with a mean of 0 and variances σ_1^2, and σ_{2a}^2 and σ_{2b}^2 respectively. All error terms and random intercepts were assumed to be independent of each other. If y had values below the limit of detection we assumed y to be left-censored at the lowest detected value of the batch.

From this model we calculated the following intraclass correlation coefficient

$$\rho = Corr(log(y_{ijk}), log(y_{i'jk})) = \frac{\sigma_{2a}^2}{\sigma_1^2 + \sigma_{2a}^2} \quad (2)$$

This ICC corresponds to the correlation between measurements i and i' from the same subject j, or the proportion of variance that is explained by differences in measurements between-subject. We used the default weakly informative priors from brms and calculated the median ICCs from draws of the posterior of every compound. This ensured that the estimates were representative of the joint posterior if the posterior of the parameters were correlated. Both models ran for 4000 iterations each with four chains, with the default number of burn in samples (i.e. 2000 in this case). The adapt delta parameter was set to 0.90. All (reported) correlations/ICCs are on the natural logarithm scale.

Table S1a: identified exogenous compounds, and associated information. The serum sample detection refers to the percentage of serum samples where the measured value was above the limited of detection. MZ = mass-to-charge ratio; RT = retention time; pos = positive; neg = negative; PFAS = per-and polyfluoroalkyl substances;

**Compound name**	**PubChem CID**	**InChl**	**Class**	**Instrument**	**Confirmed MZ**	**Confirmed RT**	**Confirmed adduct**	**Serum sample detection %**
Aldicarb-sulfone	9570093	YRRKLBAKDXSTNC-WEVVVXLNSA-N	Pesticides	C18 pos.	240.1013	53.90000	M+NH4	99.04
3-Hydroxycarbofuran	27975	RHSUJRQZTQNSLL-UHFFFAOYSA-N	Pesticides	C18 pos.	238.1074	152.49000	M+H	96.50
Propoxur	4944	ISRUGXGCCGIOQO-UHFFFAOYSA-N	Pesticides	C18 pos.	210.1125	235.15000	M+H	54.30
Isoprocarb	17517	QBSJMKIUCUGGNG-UHFFFAOYSA-N	Pesticides	C18 pos.	194.1176	253.71000	M+H	44.27
Forchlorfenuron	93379	GPXLRLUVLMHHIK-UHFFFAOYSA-N	Pesticides	C18 pos.	248.0585	244.97000	M+H	100.00
Fenobucarb	19588	DIRFUJHNVNOBMY-UHFFFAOYSA-N	Pesticides	C18 pos.	208.1332	267.88000	M+H	55.89
Cycluron	16554	DQZCVNGCTZLGAQ-UHFFFAOYSA-N	Pesticides	C18 pos.	199.1805	243.98000	M+H	78.34
Acetamiprid	213021	WCXDHFDTOYPNIE-UHFFFAOYSA-N	Pesticides	C18 pos.	223.0745	194.56758	M+H	97.61
Furalaxyl	42504	CIEXPHRYOLIQQD-UHFFFAOYSA-N	Pesticides	C18 pos.	302.1387	268.90565	M+H	82.64
Spiroxamine.1	86160	PUYXTUJWRLOUCW-UHFFFAOYSA-N	Pesticides	C18 pos.	298.2741	227.84000	M+H	99.36
Prochloraz	73665	TVLSRXXIMLFWEO-UHFFFAOYSA-N	Pesticides	C18 pos.	376.0381	261.81735	M+H	96.97
Propamocarb	32490	WZZLDXDUQPOXNW-UHFFFAOYSA-N	Pesticides	C18 pos.	189.1598	23.95000	M+H	99.84
DEET	4284	MMOXZBCLCQITDF-UHFFFAOYSA-N	Pesticides	C18 pos.	192.1383	240.64195	M+H	100.00
2-Isopropyl-6-methyl-4-pyrimidinol	135444498	AJPIUNPJBFBUKK-UHFFFAOYSA-N	Pesticides	C18 pos.	153.1022	35.18444	M+H	70.38
Perfluoroheptanesulfonic acid	67820	OYGQVDSRYXATEL-UHFFFAOYSA-N	PFAS	C18 neg.	448.9334	250.50296	M-H	98.57
Perfluorooctanoic acid	9554	SNGREZUHAYWORS-UHFFFAOYSA-N	PFAS	C18 neg.	412.9664	234.19000	M-H	100.00
Perfluorotetradecanoic acid	67822	RUDINRUXCKIXAJ-UHFFFAOYSA-N	PFAS	C18 neg.	712.9473	290.24272	M-H	46.02
Perfluorotridecanoic acid	3018355	LVDGGZAZAYHXEY-UHFFFAOYSA-N	PFAS	C18 neg.	662.9505	280.76000	M-H	43.95
Perfluorodecanoic acid	9555	PCIUEQPBYFRTEM-UHFFFAOYSA-N	PFAS	C18 neg.	512.9600	252.62000	M-H	75.48
Perfluorohexanesulfonic acid	67734	QZHDEAJFRJCDMF-UHFFFAOYSA-N	PFAS	C18 neg.	398.9366	238.98099	M-H	73.89
Perfluoroheptanoic acid	67818	ZWBAMYVPMDSJGQ-UHFFFAOYSA-N	PFAS	C18 neg.	362.9696	223.12000	M-H	68.47

Perfluorononanoic acid	67821	UZUFPBIDKMEQEQ-UHFFFAOYSA-N	PFAS	C18 neg.	462.9632	243.36000	M-H	99.36
Perfluoropropanoic acid	62356	LRMSQVBRUNSOJL-UHFFFAOYSA-N	PFAS	C18 neg.	162.9824	35.34000	M-H	98.73
Perfluorobutanesulfonic acid	67815	JGTNAGYHADQMCM-UHFFFAOYSA-N	PFAS	C18 neg.	298.9430	212.63000	M-H	42.04
Perfluorooctanesulfonic acid	74483	YFSUTJLHUFNCNZ-UHFFFAOYSA-N	PFAS	C18 neg.	498.9302	257.40612	M-H	99.68
Perfluoroundecanoic acid	77222	SIDINRCMMRKXGQ-UHFFFAOYSA-N	PFAS	C18 neg.	562.9568	261.10000	M-H	92.36
Perfluorooctanesulfonyl fluoride	9388	BHFJBHMTEDLICO-UHFFFAOYSA-N	PFAS	C18 neg.	500.9259	243.20000	M-H	99.36
N-Methylperfluorooctanesulfonamidoacetic acid	22286931	QNDHIRFIMVNHBN-UHFFFAOYSA-N	PFAS	C18 neg.	569.9674	258.72000	M-H	99.84
2-Aminohexafluoropropan-2-ol	2782456	KENSSLIXPGVLRV-UHFFFAOYSA-N	PFAS	C18 pos.	225.0457	25.70000	M+ACN+H	88.54
4-tert-Octylphenol	8814	ISAVYTVYFVQUDY-UHFFFAOYSA-N	Phenol	C18 neg.	205.1598	311.06000	M-H	100.00
Pentachlorophenol	992	IZUPBVBPLAPZRR-UHFFFAOYSA-N	Phenol	C18 neg.	262.8397	229.87000	M-H	63.54
Bisphenol S	6626	VPWNQTHUCYMVMZ-UHFFFAOYSA-N	Phenol	C18 neg.	249.0227	192.48000	M-H	67.36
Monoisononyl phthalate	110394	RNCMBSSLYOAVRT-UHFFFAOYSA-N	Phthalate	C18 neg.	291.1602	230.73000	M-H	51.59
Monoethyl phosphate	74190	ZJXZSIYSNXKHEA-UHFFFAOYSA-N	Other	C18 neg.	125.0009	18.58000	M-H	99.84
Mono-2-heptyl phthalate	5156745	NTIWNFLUZQSLDR-UHFFFAOYSA-N	Phthalate	C18 neg.	263.1289	213.86000	M-H	36.15
Monobutyl phthalate	8575	YZBOVSFWWNVKRJ-UHFFFAOYSA-N	Phthalate	C18 pos.	223.0965	242.10000	M+H	99.20
Monocyclohexyl phthalate	165618	PMDKYLLIOLFQPO-UHFFFAOYSA-N	Phthalate	C18 pos.	249.1121	252.89944	M+H	100.00
4-Hydroxybenzoic acid	135	FJKROLUGYXJWQN-UHFFFAOYSA-N	Phenol	C18 neg.	137.0244	18.25000	M-H	90.61

Table S1b: identified endogenous compounds, and associated information. The serum sample detection refers to the percentage of serum samples where the measured value was above the limit of detection. MZ = mass-to-charge ratio, RT = retention time, pos = positive, neg = negative.

Compound name	PubChem CID	InChI	Class	Instrument	Confirmed MZ	Confirmed RT	Confirmed adduct	Serum sample detection %
Dehydrolithocholic acid	5283906	KIQFUORWRVZTHT-OPTMKGCMSA-N	Bile acid	C18 neg.	373.27480	301.90000	M-H	64.81
Nordeoxycholic acid	193905	PLRQOCVIINWCFA-AHFDLSHQSA-N	Bile acid	C18 neg.	377.26970	229.80000	M-H	93.79
7-Ketochenodeoxycholate	53477693	DXOCDBGWDZAYRQ-QPVZPPSOSA-N	Bile acid	C18 neg.	389.26970	234.00000	M-H	99.84
Glycoursodeoxycholic acid	12310288	GHCZAUBVMUEKKP-XROMFQGDSA-N	Bile acid	C18 neg.	448.30680	210.50000	M-H	99.20
Glycohyodeoxcholic acid	114611	SPOIYSFQOFYOFZ-BRDORRHWSA-N	Bile acid	C18 neg.	448.30680	211.10000	M-H	99.20
Taurolithocholic acid	53477716	QBYUNVOYXHFVKC-LVMSMGIASA-N	Bile acid	C18 neg.	482.29460	242.30000	M-H	57.48
Taurodeoxycholic acid	2733768	AWDRATDZQPNJFN-VAYUFCLWSA-N	Bile acid	C18 neg.	498.28950	227.80000	M-H	96.50
Taurochenodesoxycholic acid	387316	BHTRKEVKTKCXOH-BJLOMENOSA-N	Bile acid	C18 neg.	498.28950	225.10000	M-H	96.50
Taurocholic acid	440567	WBWWGRHZICKQGZ-BKXUXKPJSA-N	Bile acid	C18 neg.	514.28440	214.20000	M-H	93.31
7-Ketodeoxycholic acid	188292	RHCPKKNRWFXMAT-RRWYKFPJSA-N	Bile acid	C18 pos.	389.26922	243.31936	M+H-H2O	99.52
Glycocholic acid	23617285	RFDAIACWWDREDC-MZMBZMQMSA-N	Bile acid	C18 pos.	448.30634	238.09531	M+H-H2O	99.84
Gama-Muricholic acid	92805	DKPMWHFRUGMUKF-KWXDGCAGSA-N	Bile acid	HILIC pos	409.29490	72.60000	M+H	37.96
Alpha-Muricholic acid	53477700	DKPMWHFRUGMUKF-LPMPYYIQSA-N	Bile acid	HILIC pos	450.32140	72.90000	M+ACN+H	99.52
Tauroursodeoxycholic acid	12443252	BHTRKEVKTKCXOH-VSHSPWMTSA-N	Bile acid	HILIC pos	500.30400	55.40000	M+H	94.26
Decanoyl-L-carnitine	10245190	LZOSYCMHQXPBFU-UHFFFAOYSA-N	Carnitine	C18 neg.	314.23370	242.20000	M-H	67.36
L-Cartinine	2724480	PHIQHXFUZVPYII-LURJTMIESA-N	Carnitine	C18 pos.	162.11250	18.70000	M+H	100.00
Acetyl-DL-carnitine	7045767	RDHQFKQIGNGIED-MRVPVSSYSA-N	Carnitine	C18 pos.	204.12303	19.97000	M+H	100.00
Propionyl-L-carnitine	107738	UFAHZIUFPNSHSL-UHFFFAOYSA-N	Carnitine	C18 pos.	218.13868	21.55000	M+H	99.52
Propanoylcarnitine	188824	UFAHZIUFPNSHSL-MRVPVSSYSA-N	Carnitine	C18 pos.	218.13868	22.26000	M+H	99.52
Butanoylcarnitine	213144	QWYFHHGCZUCMBN-SECBINFHSA-N	Carnitine	C18 pos.	232.15433	31.30000	M+H	100.00
Isobutyryl-L-carnitine	168379	LRCNOZRCYBNMEP-SECBINFHSA-N	Carnitine	C18 pos.	232.15433	30.53937	M+H	100.00

Tiglylcarnitine	91825636	WURBQCVBQNMUQT-OLKPEBQYSA-N	Carnitine	C18 pos.	244.15433	38.45092	M+H	99.68
2-Methylbutyroylcarnitine	6426901	IHCPDBBYTYJYIL-UHFFFAOYSA-N	Carnitine	C18 pos.	246.16998	43.56489	M+H	100.00
Hydroxybutyrylcarnitine	90659885	UEFRDQSMQXDWTO-YGPZHTELSA-N	Carnitine	C18 pos.	248.14925	19.78000	M+H	99.36
Hexanoylcarnitine	3246938	VVPRQWTYSNDTEA-LLVKDONJSA-N	Carnitine	C18 pos.	260.18563	121.00000	M+H	100.00
Succinylcarnitine	71464481	HAEVNYBCYZZDFL-UHFFFAOYSA-N	Carnitine	C18 pos.	262.12851	23.20167	M+H	61.31
Octanoyl-L-carnitine	11953814	CXTATJFJDMJMIY-CYBMUJFWSA-N	Carnitine	C18 pos.	288.21693	210.57000	M+H	99.04
Dodecenoylcarnitine	53481671	JEOZLTJHDSKQIT-CLVCIHKQSA-N	Carnitine	C18 pos.	342.26388	242.10467	M+H	99.84
3-hydroxydodecanoyl carnitine	71464535	ULWDPUHFMOBGFJ-UHFFFAOYSA-N	Carnitine	C18 pos.	360.27445	228.53000	M+H	98.57
Tetradecenoyl-L-carnitine	53481677	VDPVQRIJJXMKLV-VVLLFNJHSA-N	Carnitine	C18 pos.	370.29518	259.91144	M+H	99.36
Myristoyl-L-carnitine	53477791	PSHXNVGSVNEJBD-LJQANCHMSA-N	Carnitine	C18 pos.	372.31083	263.93828	M+H	99.84
3-Hydroxyhexadecanoylcarnitine	71464553	XKAZIAFZAQAHHG-UHFFFAOYSA-N	Carnitine	C18 pos.	416.33705	259.48000	M+H	99.04
Linoleyl carnitine	6450015	MJLXQSQYKZWZCB-DQFWFXSYSA-N	Carnitine	C18 pos.	424.34213	271.24000	M+H	99.84
Oleoylcarnitine	46907933	IPOLTUVFXFHAHI-WHIOSMTNSA-N	Carnitine	C18 pos.	426.35778	281.21000	M+H	99.84
Octadecanoylcarnitine	52922056	FNPHNLNTJNMAEE-HSZRJFAPSA-N	Carnitine	C18 pos.	428.37344	295.74218	M+H	99.52
Isovaleryl-L-carnitine	6426851	IGQBPDJNUXPEMT-UHFFFAOYSA-N	Carnitine	HILIC pos	246.17000	230.20000	M+H	100.00
Valeryl-L-carnitine	53481619	VSNFQQXVMPSASB-JTQLQIEISA-N	Carnitine	HILIC pos	246.17000	230.20000	M+H	100.00
Adipoyl-L-carnitine	71296139	BSVHAXJKBCWVDA-UHFFFAOYSA-N	Carnitine	HILIC pos	290.15980	250.00000	M+H	100.00
Methylglutaryl-L-carnitine	128145	HFCPFJNSBPQJDP-UHFFFAOYSA-N	Carnitine	HILIC pos	290.15980	248.20000	M+H	100.00
Acetoacetate; Acetoacetic acid	96	WDJHALXBUFZDSR-UHFFFAOYSA-N	Endogenous metabolite	C18 neg.	101.02440	19.70000	M-H	99.84
N-Formylglycine	75606	UGJBHEZMOKVTIM-UHFFFAOYSA-N	Endogenous metabolite	C18 neg.	102.01970	19.30000	M-H	37.26
Aminoisobutanoate	64956	QCHPKSFMDHPSNR-UHFFFAOYSA-N	Endogenous metabolite	C18 neg.	102.05610	20.80000	M-H	99.52
Malonate	867	OFOBLEOULBTSOW-UHFFFAOYSA-N	Endogenous metabolite	C18 neg.	103.00370	19.00000	M-H	100.00
Hydroxypyruvate	964	HHDDCCUIIUWNGJ-UHFFFAOYSA-N	Endogenous metabolite	C18 neg.	103.00370	19.40000	M-H	100.00

2-Hydroxybutyrate	440864	AFENDNXGAFYKQO-VKHMYHEASA-N	Endogenous metabolite	C18 neg.	103.04010	19.80000	M-H	92.83
Serine	5951	MTCFGRXMJLQNBG-REOHCLBHSA-N	Endogenous metabolite	C18 neg.	104.03530	20.80000	M-H	100.00
2-Aminophenol	5801	CDAWCLOXVUBKRW-UHFFFAOYSA-N	Endogenous metabolite	C18 neg.	108.04550	20.90000	M-H	97.13
Pyrocatechol	289	YCIMNLLNPGFGHC-UHFFFAOYSA-N	Endogenous metabolite	C18 neg.	109.02950	20.00000	M-H	99.84
Hydroquinone	785	QIGBRXMKCJKVMJ-UHFFFAOYSA-N	Endogenous metabolite	C18 neg.	109.02950	22.40000	M-H	99.84
2,6-Dihydroxypyridine	69371	WLFXSECCHULRRO-UHFFFAOYSA-N	Endogenous metabolite	C18 neg.	110.02480	19.80000	M-H	55.10
Pyrrole-2-Carboxylate	12473	WRHZVMBBRYBTKZ-UHFFFAOYSA-N	Endogenous metabolite	C18 neg.	110.02480	20.20000	M-H	55.10
Uracil	1174	ISAKRJDGNUQOIC-UHFFFAOYSA-N	Endogenous metabolite	C18 neg.	111.02000	23.00000	M-H	98.89
Creatinine	588	DDRJAANPRJIHGJ-UHFFFAOYSA-N	Endogenous metabolite	C18 neg.	112.05160	22.00000	M-H	100.00
Proline	145742	ONIBWKKTOPOVIA-BYPYZUCNSA-N	Endogenous metabolite	C18 neg.	114.05610	20.80000	M-H	100.00
Maleate	444266	VZCYOOQTPOCHFL-UPHRSURJSA-N	Endogenous metabolite	C18 neg.	115.00370	19.80000	M-H	99.84
N-Acetylglycine	10972	OKJIRPAQVSHGFK-UHFFFAOYSA-N	Endogenous metabolite	C18 neg.	116.03530	19.70000	M-H	97.93
Valine	6287	KZSNJWFQEVHDMF-BYPYZUCNSA-N	Endogenous metabolite	C18 neg.	116.07170	21.90000	M-H	99.84
Norvaline	439575	SNDPXSYFESPGGJ-SCSAIBSYSA-N	Endogenous metabolite	C18 neg.	116.07170	21.40000	M-H	99.84
5-Aminopentanoate	138	JJMDCOVWQOJGCB-UHFFFAOYSA-N	Endogenous metabolite	C18 neg.	116.07170	20.10000	M-H	99.84
Succinate; Succinic acid	1110	KDYFGRWQOYBRFD-UHFFFAOYSA-N	Endogenous metabolite	C18 neg.	117.01930	17.90000	M-H	98.25
Methylmalonate; Methylmalonic acid	487	ZIYVHBGGAOATLY-UHFFFAOYSA-N	Endogenous metabolite	C18 neg.	117.01930	19.50000	M-H	98.25

Homoserine	12647	UKAUYVFTDYCKQA-VKHMYHEASA-N	Endogenous metabolite	C18 neg.	118.05100	20.40000	M-H	100.00
Benzoate	243	WPYMKLBDIGXBTP-UHFFFAOYSA-N	Endogenous metabolite	C18 neg.	121.02950	27.30000	M-H	73.89
Nicotinate	938	PVNIIMVLHYAWGP-UHFFFAOYSA-N	Endogenous metabolite	C18 neg.	122.02480	20.10000	M-H	100.00
3-Hydroxybenzyl Alcohol	102	OKVJCVWFVRATSG-UHFFFAOYSA-N	Endogenous metabolite	C18 neg.	123.04520	57.30000	M-H	98.57
Taurine	1123	XOAAWQZATWQOTB-UHFFFAOYSA-N	Endogenous metabolite	C18 neg.	124.00740	20.30000	M-H	100.00
5-Methylcytosine	65040	LRSASMSXMSNRBT-UHFFFAOYSA-N	Endogenous metabolite	C18 neg.	124.05160	23.20000	M-H	42.04
Thymine	1135	RWQNBRDOKXIBIV-UHFFFAOYSA-N	Endogenous metabolite	C18 neg.	125.03570	26.40000	M-H	88.54
4-Imidazoleacetate	96215	PRJKNHOMHKJCEJ-UHFFFAOYSA-N	Endogenous metabolite	C18 neg.	125.03570	20.40000	M-H	88.54
Oxoproline	439685	ODHCTXKNWHHXJC-GSVOUGTGSA-N	Endogenous metabolite	C18 neg.	128.03530	19.20000	M-H	97.93
Pyroglutamate; 5-Oxoproline	7405	ODHCTXKNWHHXJC-VKHMYHEASA-N	Endogenous metabolite	C18 neg.	128.03530	19.20000	M-H	97.93
Itaconate	811	LVHBHZANLOWSRM-UHFFFAOYSA-N	Endogenous metabolite	C18 neg.	129.01930	18.30000	M-H	100.00
2-Methylmaleate; Citraconic acid	643798	HNEGQIOMVPPMNR-IHWYPQMZSA-N	Endogenous metabolite	C18 neg.	129.01930	18.50000	M-H	100.00
3-Methyl-2-Oxovalerate	47	JVQYSWDUAOAHFM-UHFFFAOYSA-N	Endogenous metabolite	C18 neg.	129.05570	26.40000	M-H	99.84
Ketoleucine	70	BKAJNAXTPSGJCU-UHFFFAOYSA-N	Endogenous metabolite	C18 neg.	129.05570	31.00000	M-H	99.84
Trans-4-Hydroxy-L-Proline	69248	PMMYEEVYMWASQN-BKLSDQPFSA-N	Endogenous metabolite	C18 neg.	130.05100	20.40000	M-H	99.52
5-Aminolevulinate; 5-Aminolevulinic acid	137	ZGXJTSGNIOSYLO-UHFFFAOYSA-N	Endogenous metabolite	C18 neg.	130.05100	20.40000	M-H	99.52
N-Acetylalanine	88064	KTHDTJVBEPMMGL-VKHMYHEASA-N	Endogenous metabolite	C18 neg.	130.05100	19.40000	M-H	99.52

Isoleucine	6306	AGPKZVBTJJNPAG-WHFBIAKZSA-N	Endogenous metabolite	C18 neg.	130.08740	23.90000	M-H	100.00
Norleucine	21236	LRQKBLKVPFOOQJ-YFKPBYRVSA-N	Endogenous metabolite	C18 neg.	130.08740	25.00000	M-H	100.00
Glutarate; Glutaric acid	743	JFCQEDHGNNZCLN-UHFFFAOYSA-N	Endogenous metabolite	C18 neg.	131.03500	18.40000	M-H	95.70
Ethylmalonate	11756	UKFXDFUAPNAMPJ-UHFFFAOYSA-N	Endogenous metabolite	C18 neg.	131.03500	19.70000	M-H	95.70
Asparagine	6267	DCXYFEDJOCDNAF-REOHCLBHSA-N	Endogenous metabolite	C18 neg.	131.04620	20.50000	M-H	98.73
D-Ornithine	71082	AHLPHDHHMVZTML-SCSAIBSYSA-N	Endogenous metabolite	C18 neg.	131.08260	21.80000	M-H	100.00
L-Ornithine	6262	AHLPHDHHMVZTML-BYPYZUCNSA-N	Endogenous metabolite	C18 neg.	131.08260	21.80000	M-H	100.00
Aspartate	5960	CKLJMWTZIZZHCS-REOHCLBHSA-N	Endogenous metabolite	C18 neg.	132.03020	18.90000	M-H	100.00
Malate	92824	BJEPYKJPYRNKOW-UWTATZPHSA-N	Endogenous metabolite	C18 neg.	133.01420	18.20000	M-H	99.84
Urocanate	736715	LOIYMIARKYCTBW-OWOJBTEDSA-N	Endogenous metabolite	C18 neg.	137.03570	19.60000	M-H	87.58
3-Methylglutaconate	1551553	WKRBKYFIJPGYQC-DUXPYHPUSA-N	Endogenous metabolite	C18 neg.	143.03500	18.50000	M-H	100.00
Oxoglutarate; 2-Oxoglutaric acid	51	KPGXRSRHYNQIFN-UHFFFAOYSA-N	Endogenous metabolite	C18 neg.	145.01420	18.10000	M-H	96.34
Methyglutarate; 3-Methylglutaric acid	12284	XJMMNTGIMDZPMU-UHFFFAOYSA-N	Endogenous metabolite	C18 neg.	145.05060	18.50000	M-H	93.79
2-Methylglutarate	12046	AQYCMVICBNBXNA-UHFFFAOYSA-N	Endogenous metabolite	C18 neg.	145.05060	18.30000	M-H	93.79
Adipate; Adipic acid	196	WNLRTRBMVRJNCN-UHFFFAOYSA-N	Endogenous metabolite	C18 neg.	145.05060	18.00000	M-H	93.79
Glutamine	5961	ZDXPYRJPNDTMRX-VKHMYHEASA-N	Endogenous metabolite	C18 neg.	145.06190	20.40000	M-H	100.00
Lysine	5962	KDXKERNSBIXSRK-YFKPBYRVSA-N	Endogenous metabolite	C18 neg.	145.09830	22.00000	M-H	100.00

Glutamate	33032	WHUUTDBJXJRKMK-VKHMYHEASA-N	Endogenous metabolite	C18 neg.	146.04590	18.60000	M-H	99.84
Citramalate	1081	XFTRTWQBIOMVPK-UHFFFAOYSA-N	Endogenous metabolite	C18 neg.	147.02990	18.60000	M-H	99.84
Trans-Cinnamate	444539	WBYWAXJHAXSJNI-VOTSOKGWSA-N	Endogenous metabolite	C18 neg.	147.04520	50.60000	M-H	99.84
Mevalonate	439230	KJTLQQUUPVSXIM-ZCFIWIBFSA-N	Endogenous metabolite	C18 neg.	147.06630	19.40000	M-H	94.59
Methionine	6137	FFEARJCKVFRZRR-BYPYZUCNSA-N	Endogenous metabolite	C18 neg.	148.04380	23.20000	M-H	98.73
Tartrate	439655	FEWJPZIEWOKRBE-LWMBPPNESA-N	Endogenous metabolite	C18 neg.	149.00920	18.30000	M-H	72.29
Xylose	135191	SRBFZHDQGSBBOR-IOVATXLUSA-N	Endogenous metabolite	C18 neg.	149.04550	20.70000	M-H	100.00
Arabinose	66308	PYMYPHUHKUWMLA-WDCZJNDASA-N	Endogenous metabolite	C18 neg.	149.04550	20.10000	M-H	100.00
Lyxose	439240	SRBFZHDQGSBBOR-AGQMPKSLSA-N	Endogenous metabolite	C18 neg.	149.04550	19.50000	M-H	100.00
Xanthine	1188	LRFVTYWOQMYALW-UHFFFAOYSA-N	Endogenous metabolite	C18 neg.	151.02610	22.20000	M-H	98.73
2',4'-Dihydroxyacetophenone	6990	SULYEHHGGXARJS-UHFFFAOYSA-N	Endogenous metabolite	C18 neg.	151.04010	191.70000	M-H	88.38
P-Hydroxyphenylacetate; 4-Hydroxyphenylacetic acid	127	XQXPVVBIMDBYFF-UHFFFAOYSA-N	Endogenous metabolite	C18 neg.	151.04010	19.50000	M-H	98.57
Mandelate	439616	IWYDHOAUDWTVEP-ZETCQYMHSA-N	Endogenous metabolite	C18 neg.	151.04010	26.20000	M-H	98.57
2,3-Dihydroxybenzoate	19	GLDQAMYCGOIJDV-UHFFFAOYSA-N	Endogenous metabolite	C18 neg.	153.01930	26.30000	M-H	100.00
Histidine	6274	HNDVDQJCIGZPNO-YFKPBYRVSA-N	Endogenous metabolite	C18 neg.	154.06220	22.10000	M-H	100.00
Orotate	967	PXQPEWDEAKTCGB-UHFFFAOYSA-N	Endogenous metabolite	C18 neg.	155.00980	19.20000	M-H	97.77
N-Acetylproline	66141	GNMSLDIYJOSUSW-LURJTMIESA-N	Endogenous metabolite	C18 neg.	156.06660	20.40000	M-H	100.00

Oxoadipate; 2-Oxoadipic acid	71	FGSBNBBHOZHUBO-UHFFFAOYSA-N	Endogenous metabolite	C18 neg.	159.02990	18.30000	M-H	99.52
6-Carboxyhexanoate	385	WLJVNTCWHIRURA-UHFFFAOYSA-N	Endogenous metabolite	C18 neg.	159.06630	18.80000	M-H	86.46
Aminoadipate	92136	OYIFNHCXNCRBQI-BYPYZUCNSA-N	Endogenous metabolite	C18 neg.	160.06150	18.90000	M-H	99.68
N-Methylglutamate	439377	XLBVNMSMFQMKEY-BYPYZUCNSA-N	Endogenous metabolite	C18 neg.	160.06150	19.30000	M-H	99.68
3-Hydroxymethylglutarate; 3-Hydroxy-3-methylglutaric acid	1662	NPOAOTPXWNWTSH-UHFFFAOYSA-N	Endogenous metabolite	C18 neg.	161.04550	18.30000	M-H	98.41
Phenylpyruvate	997	BTNMPGBKDVTSJY-UHFFFAOYSA-N	Endogenous metabolite	C18 neg.	163.04010	41.00000	M-H	99.68
2-Deoxy-D-Glucose	439268	PMMURAAUARKVCB-CERMHHMHSA-N	Endogenous metabolite	C18 neg.	163.06120	20.70000	M-H	96.02
Fucose	17106	SHZGCJCMOBCMKK-DHVFOXMCSA-N	Endogenous metabolite	C18 neg.	163.06120	21.20000	M-H	96.02
Rhamnose	25310	SHZGCJCMOBCMKK-JFNONXLTSA-N	Endogenous metabolite	C18 neg.	163.06120	21.40000	M-H	96.02
3-(2-Hydroxyphenyl)Propanoate	873	CJBDUOMQLFKVQC-UHFFFAOYSA-N	Endogenous metabolite	C18 neg.	165.05570	57.30000	M-H	100.00
Quinolinate; Quinolinic acid	1066	GJAWHXHKYYXBSV-UHFFFAOYSA-N	Endogenous metabolite	C18 neg.	166.01460	19.60000	M-H	96.02
4-Hydroxy-L-Phenylglycine	36143	LJCWONGJFPCTTL-ZETCQYMHSA-N	Endogenous metabolite	C18 neg.	166.05100	21.60000	M-H	89.17
Pyridoxal	1050	RADKZDMFGJYCBB-UHFFFAOYSA-N	Endogenous metabolite	C18 neg.	166.05100	23.50000	M-H	89.17
Urate	1175	LEHOTFFKMJEONL-UHFFFAOYSA-N	Endogenous metabolite	C18 neg.	167.02110	19.50000	M-H	99.68
Pyridoxamine	1052	NHZMQXZHNVQTQA-UHFFFAOYSA-N	Endogenous metabolite	C18 neg.	167.08260	23.10000	M-H	36.94
3-Methyl-L-Histidine; 1-Methyl-L-Histidine	64969	JDHILDINMRGULE-LURJTMIESA-N	Endogenous metabolite	C18 neg.	168.07790	24.10000	M-H	62.26
N-Acetylleucine	70912	WXNXCEHXYPACJF-ZETCQYMHSA-N	Endogenous metabolite	C18 neg.	172.09790	24.90000	M-H	94.90

Trans-Aconitate	444212	GTZCVFVGUGFEME-HNQUOIGGSA-N	Endogenous metabolite	C18 neg.	173.00920	17.40000	M-H	100.00
Shikimate	8742	JXOHGGNKMLTUBP-HSUXUTPPSA-N	Endogenous metabolite	C18 neg.	173.04550	19.30000	M-H	97.77
N-Acetylasparagine	99715	HXFOXFJUNFFYMO-BYPYZUCNSA-N	Endogenous metabolite	C18 neg.	173.05680	19.50000	M-H	72.29
Suberate	10457	TYFQFVWCELRYAO-UHFFFAOYSA-N	Endogenous metabolite	C18 neg.	173.08190	18.70000	M-H	90.92
Arginine	6322	ODKSFYDXXFIFQN-BYPYZUCNSA-N	Endogenous metabolite	C18 neg.	173.10440	22.30000	M-H	100.00
N-Acetylaspartate	65065	OTCCIMWXFLJLIA-BYPYZUCNSA-N	Endogenous metabolite	C18 neg.	174.04080	18.40000	M-H	99.84
Indole-3-Acetate	802	SEOVTRFCIGRIMH-UHFFFAOYSA-N	Endogenous metabolite	C18 neg.	174.05610	47.40000	M-H	98.57
Citrulline	9750	RHGKLRLOHDJJDR-BYPYZUCNSA-N	Endogenous metabolite	C18 neg.	174.08840	20.30000	M-H	100.00
Ascorbate	54670067	CIWBSHSKHKDKBQ-JLAZNSOCSA-N	Endogenous metabolite	C18 neg.	175.02480	19.10000	M-H	89.49
2-Keto-3-Deoxy-D-Gluconic Acid	194024	WPAMZTWLKIDIOP-NQXXGFSBSA-N	Endogenous metabolite	C18 neg.	177.04050	19.00000	M-H	98.41
Hippurate; Hippuric acid	464	QIAFMBKCNZACKA-UHFFFAOYSA-N	Endogenous metabolite	C18 neg.	178.05100	28.00000	M-H	99.84
Caffeate	1549111	QAIPRVGONGVQAS-RQOWECAXSA-N	Endogenous metabolite	C18 neg.	179.03500	24.00000	M-H	95.86
Gulose	10130220	WQZGKKKJIJFFOK-QRXFDPRISA-N	Endogenous metabolite	C18 neg.	179.05610	20.80000	M-H	100.00
Psicose	441036	LKDRXBCSQODPBY-JDJSBBGDSA-N	Endogenous metabolite	C18 neg.	179.05610	21.10000	M-H	100.00
Tyrosine	6057	OUYCCCASQSFEME-QMMMGPOBSA-N	Endogenous metabolite	C18 neg.	180.06660	23.30000	M-H	99.84
Epinephrine	5816	UCTWMZQNUQWSLP-VIFPVBQESA-N	Endogenous metabolite	C18 neg.	182.08230	25.30000	M-H	41.40
Phosphorylcholine	1014	YHHSONZFOIEMCP-UHFFFAOYSA-O	Endogenous metabolite	C18 neg.	183.06660	25.40000	M-H	97.29

2-Phosphoglycerate	59	GXIURPTVHJPJLF-UHFFFAOYSA-N	Endogenous metabolite	C18 neg.	184.98570	17.60000	M-H	40.76
N-Alpha-Acetyllysine	192590	VEYYWZRYIYDQJM-UHFFFAOYSA-N	Endogenous metabolite	C18 neg.	187.10880	20.50000	M-H	33.92
Kynurenate; Kynurenic acid	3845	HCZHHEIFKROPDY-UHFFFAOYSA-N	Endogenous metabolite	C18 neg.	188.03530	27.40000	M-H	96.02
N-Acetylglutamate	70914	RFMMMVDNIPUKGG-YFKPBYRVSA-N	Endogenous metabolite	C18 neg.	188.05640	18.00000	M-H	100.00
5-Hydroxyindoleacetate	1826	DUUGKQCEGZLZNO-UHFFFAOYSA-N	Endogenous metabolite	C18 neg.	190.05100	21.80000	M-H	99.84
N-Acetylmethionine	6180	XUYPXLNMDZIRQH-UHFFFAOYSA-N	Endogenous metabolite	C18 neg.	190.05430	20.50000	M-H	44.75
Isocitrate	1198	ODBLHEXUDAPZAU-UHFFFAOYSA-N	Endogenous metabolite	C18 neg.	191.01970	17.70000	M-H	100.00
Glucuronate	444791	AEMOLEFTQBMNLQ-WAXACMCWSA-N	Endogenous metabolite	C18 neg.	193.03540	19.00000	M-H	53.82
Ferulate	445858	KSEBMYQBYZTDHS-HWKANZROSA-N	Endogenous metabolite	C18 neg.	193.05060	27.90000	M-H	81.21
Gluconate	10690	RGHNJXZEOKUKBD-SQOUGZDYSA-N	Endogenous metabolite	C18 neg.	195.05100	19.20000	M-H	100.00
Laurate	3893	POULHZVOKOAJMA-UHFFFAOYSA-N	Endogenous metabolite	C18 neg.	199.17040	289.60000	M-H	83.12
Sebacate	5192	CXMXRPHRNRROMY-UHFFFAOYSA-N	Endogenous metabolite	C18 neg.	201.11320	19.50000	M-H	99.84
2-Oxobutanoate; 2-Oxobutyric acid	58	TYEYBOSBBBHJIV-UHFFFAOYSA-N	Endogenous metabolite	C18 neg.	203.05610	19.80000	2M-H	99.84
Tryptophan	9060	QIVBCDIJIAJPQS-SECBINFHSA-N	Endogenous metabolite	C18 neg.	203.08260	36.20000	M-H	100.00
2-Methylcitrate; 2-Methylcitric acid	515	YNOXCRMFGMSKIJ-UHFFFAOYSA-N	Endogenous metabolite	C18 neg.	205.03540	18.70000	M-H	99.52
N-Acetylphenylalanine	74839	CBQJSKKFNMDLON-JTQLQIEISA-N	Endogenous metabolite	C18 neg.	206.08230	44.60000	M-H	98.41
Kynurenine	161166	YGPSJZOEDVAXAB-QMMMGPOBSA-N	Endogenous metabolite	C18 neg.	207.07750	27.80000	M-H	99.84

Galactarate	3037582	DSLZVSRJTYRBFB-DUHBMQHGSA-N	Endogenous metabolite	C18 neg.	209.03030	18.30000	M-H	96.02
Saccharate	33037	DSLZVSRJTYRBFB-LLEIAEIESA-N	Endogenous metabolite	C18 neg.	209.03030	18.20000	M-H	96.02
D-Sedoheptulose	102926	HSNZZMHEPUFJNZ-UHFFFAOYSA-N	Endogenous metabolite	C18 neg.	209.06670	20.50000	M-H	98.89
3-Methoxytyrosine	1670	PFDUUKDQEHURQC-UHFFFAOYSA-N	Endogenous metabolite	C18 neg.	210.07720	24.10000	M-H	99.84
Indoxyl Sulfate	10258	BXFFHSIDQOFMLE-UHFFFAOYSA-N	Endogenous metabolite	C18 neg.	212.00230	54.40000	M-H	99.68
Omega-Hydroxydodecanoate	79034	ZDHCZVWCTKTBRY-UHFFFAOYSA-N	Endogenous metabolite	C18 neg.	215.16530	208.00000	M-H	100.00
O-Succinyl-Homoserine	439406	GNISQJGXJIDKDJ-YFKPBYRVSA-N	Endogenous metabolite	C18 neg.	218.06700	19.20000	M-H	53.34
5-Hydroxy-L-Tryptophan	144	LDCYZAJDBXYCGN-UHFFFAOYSA-N	Endogenous metabolite	C18 neg.	219.07750	25.70000	M-H	92.99
Cystathionine	439258	ILRYLPWNYFXEMH-WHFBIAKZSA-N	Endogenous metabolite	C18 neg.	221.06020	20.20000	M-H	99.52
Myristate	11005	TUNFSRHWOTWDNC-UHFFFAOYSA-N	Endogenous metabolite	C18 neg.	227.20170	343.40000	M-H	99.36
Lumichrome	5326566	ZJTJUVIJVLLGSP-UHFFFAOYSA-N	Endogenous metabolite	C18 neg.	241.07310	204.60000	M-H	100.00
Uridine	6029	DRTQHJPVMGBUCF-XVFCMESISA-N	Endogenous metabolite	C18 neg.	243.06230	22.60000	M-H	99.84
Palmitoleate	445638	SECPZKHBENQXJG-FPLPWBNLSA-N	Endogenous metabolite	C18 neg.	253.21730	352.40000	M-H	99.84
N-Acetylglucosamine	439174	OVRNDRQMDRJTHS-RTRLPJTCSA-N	Endogenous metabolite	C18 neg.	256.05930	20.90000	M+Cl	38.69
N-Acetylgalactosamine	84265	OVRNDRQMDRJTHS-CBQIKETKSA-N	Endogenous metabolite	C18 neg.	256.05930	21.10000	M+Cl	38.69
Inosine	6021	UGQMRVRMYYASKQ-KQYNXXCUSA-N	Endogenous metabolite	C18 neg.	267.07350	22.40000	M-H	83.92
Heptadecanoate	10465	KEMQGTRYUADPNZ-UHFFFAOYSA-N	Endogenous metabolite	C18 neg.	269.24860	397.20000	M-H	100.00

Eicosapentaenoate	446284	JAZBEHYOTPTENJ-JLNKQSITSA-N	Endogenous metabolite	C18 neg.	301.21730	361.30000	M-H	99.52
N-Acetylneuraminate	445063	SQVRNKJHWKZAKO-PFQGKNLYSA-N	Endogenous metabolite	C18 neg.	308.09870	18.90000	M-H	98.89
Lauroylcarnitine	168381	FUJLYHJROOYKRA-QGZVFWFLSA-N	Endogenous metabolite	C18 neg.	342.26500	263.90000	M-H	94.59
Lithocholate	11740284	SMEROWZSTRWXGI-YPLGJCPNSA-N	Endogenous metabolite	C18 neg.	375.29050	295.80000	M-H	93.15
Oleoyl-Glycerol	5283468	RZRNAYUHWVFMIP-KTKRTIGZSA-N	Endogenous metabolite	C18 neg.	391.26210	365.90000	M+Cl	83.44
Ursodeoxycholate	31401	RUDATBOHQWOJDD-UZVSRGJWSA-N	Endogenous metabolite	C18 neg.	391.28540	229.40000	M-H	89.81
Glycochenodeoxycholate	12544	GHCZAUBVMUEKKP-GYPHWSFCSA-N	Endogenous metabolite	C18 neg.	448.30680	229.80000	M-H	99.20
Glycocholate	10140	RFDAIACWWDREDC-FRVQLJSFSA-N	Endogenous metabolite	C18 neg.	464.30180	218.20000	M-H	98.57
Lithocholyltaurine	439763	QBYUNVOYXHFVKC-GBURMNQMSA-N	Endogenous metabolite	C18 neg.	482.29460	243.00000	M-H	57.48
L-Alanine	5950	QNAYBMKLOCPYGJ-REOHCLBHSA-N	Endogenous metabolite	C18 neg.	88.04040	20.80000	M-H	99.84
Sarcosine	1088	FSYKKLYZXJSNPZ-UHFFFAOYSA-N	Endogenous metabolite	C18 neg.	88.04040	20.90000	M-H	99.84
D-Alanine	71080	QNAYBMKLOCPYGJ-UWTATZPHSA-N	Endogenous metabolite	C18 neg.	88.04040	20.90000	M-H	99.84
Beta-Alanine	239	UCMIRNVEIXFBKS-UHFFFAOYSA-N	Endogenous metabolite	C18 neg.	88.04040	21.20000	M-H	99.84
Oxalate	971	MUBZPKHOEPUJKR-UHFFFAOYSA-N	Endogenous metabolite	C18 neg.	88.98800	18.70000	M-H	99.20
Lactate	61503	JVTAAEKCZFNVCJ-UWTATZPHSA-N	Endogenous metabolite	C18 neg.	89.02440	19.30000	M-H	99.20
Cytosine	597	OPTASPLRGRRNAP-UHFFFAOYSA-N	Endogenous metabolite	C18 pos.	112.05054	20.73180	M+H	89.97
Threonine	6288	AYFVYJQAPQTCCC-GBXIJSLDSA-N	Endogenous metabolite	C18 pos.	120.06552	22.48580	M+H	100.00

Nicotinamide	936	DFPAKSUCGFBDDF-UHFFFAOYSA-N	Endogenous metabolite	C18 pos.	123.05529	24.97261	M+H	98.25
2-Methylbutanal	7284	BYGQBDHUGHBGMD-UHFFFAOYSA-N	Endogenous metabolite	C18 pos.	128.10699	21.92798	M+ACN+H	100.00
Quinoline	7047	SMWDFEZZVXVKRB-UHFFFAOYSA-N	Endogenous metabolite	C18 pos.	130.06513	31.96801	M+H	99.84
Pipecolic acid	849	HXEACLLIILLPRG-YFKPBYRVSA-N	Endogenous metabolite	C18 pos.	130.08626	24.92300	M+H	100.00
Sorbic acid	643460	WSWCOQWTEOXDQX-MQQKCMAXSA-N	Endogenous metabolite	C18 pos.	130.08630	19.00000	M+NH4	100.00
N-Acetylputrescine	122356	KLZGKIDSEJWEDW-UHFFFAOYSA-N	Endogenous metabolite	C18 pos.	131.11789	19.18000	M+H	95.86
Dihydrouracil	649	OIVLITBTBDPEFK-UHFFFAOYSA-N	Endogenous metabolite	C18 pos.	132.07675	22.28785	M+NH4	99.84
Creatine	586	CVSVTCORWBXHQV-UHFFFAOYSA-N	Endogenous metabolite	C18 pos.	132.07676	22.28785	M+H	99.84
Hexanoate	8892	FUZZWVXGSFPDMH-UHFFFAOYSA-N	Endogenous metabolite	C18 pos.	134.11755	20.79838	M+NH4	99.52
Hypoxanthine	135398638	FDGQSTZJBFJUBT-UHFFFAOYSA-N	Endogenous metabolite	C18 pos.	137.04579	25.17233	M+H	93.31
Trigonelline	5570	WWNNZCOKKKDOPX-UHFFFAOYSA-N	Endogenous metabolite	C18 pos.	138.05496	21.94000	M+H	98.41
Tyramine	5610	DZGWFCGJZKJUFP-UHFFFAOYSA-N	Endogenous metabolite	C18 pos.	138.09134	21.99000	M+H	69.90
Phenylethanolamine	1000	ULSIYEODSMZIPX-UHFFFAOYSA-N	Endogenous metabolite	C18 pos.	138.09134	26.72927	M+H	69.90
4-Acetamidobutyric acid	18189	UZTFMUBKZQVKLK-UHFFFAOYSA-N	Endogenous metabolite	C18 pos.	146.08117	29.82088	M+H	99.20
Deoxycarnitine	725	JHPNVNIEXXLNTR-UHFFFAOYSA-O	Endogenous metabolite	C18 pos.	146.11756	22.37347	M+H	100.00
3-Methyl-2-Oxindole	150923	BBZCPUCZKLTAJQ-UHFFFAOYSA-N	Endogenous metabolite	C18 pos.	148.07569	200.23538	M+H	83.92
Norepinephrine	439260	SFLSHLFXELFNJZ-QMMMGPOBSA-N	Endogenous metabolite	C18 pos.	152.07119	23.08128	M+H-H2O	57.17

3-Amino-5-Hydroxybenzoate	127115	QPEJHSFTZVMSJH-UHFFFAOYSA-N	Endogenous metabolite	C18 pos.	154.04987	24.67000	M+H	99.84
Dopamine	681	VYFYYTLLBUKUHU-UHFFFAOYSA-N	Endogenous metabolite	C18 pos.	154.08626	23.50051	M+H	95.54
L-Carnitine	10917	PHIQHXFUZVPYII-ZCFIWIBFSA-N	Endogenous metabolite	C18 pos.	162.11247	22.18759	M+H	100.00
Phenylalanine	6140	COLNVLDHVKWLRT-QMMMGPOBSA-N	Endogenous metabolite	C18 pos.	166.08626	29.52173	M+H	99.68
3-Methoxytyramine	1669	DIVQKHQLANKJQO-UHFFFAOYSA-N	Endogenous metabolite	C18 pos.	168.10191	22.77000	M+H	87.42
Phosphoenolpyruvic acid	1005	DTBNBXWJWCWCIK-UHFFFAOYSA-N	Endogenous metabolite	C18 pos.	168.98965	30.36481	M+H	75.32
Pyridoxine	1054	LXNHXLLTXMVWPM-UHFFFAOYSA-N	Endogenous metabolite	C18 pos.	170.08117	22.84516	M+H	97.93
2-Quinolinecarboxylate	7124	LOAUVZALPPNFOQ-UHFFFAOYSA-N	Endogenous metabolite	C18 pos.	174.05496	51.24407	M+H	97.93
Indole-3-Acetamide	397	ZOAMBXDOGPRZLP-UHFFFAOYSA-N	Endogenous metabolite	C18 pos.	175.08659	115.29911	M+H	98.73
N-Methyltryptamine	6088	NCIKQJBVUNUXLW-UHFFFAOYSA-N	Endogenous metabolite	C18 pos.	175.12298	47.31249	M+H	75.80
Guanidinosuccinic acid	439918	VVHOUVWJCQOYGG-REOHCLBHSA-N	Endogenous metabolite	C18 pos.	176.06659	20.50000	M+H	92.20
Galactosamine	24154	MSWZFWKMSRAUBD-GASJEMHNSA-N	Endogenous metabolite	C18 pos.	180.08665	19.50000	M+H	91.56
Salsolinol	91588	IBRKLUSXDYATLG-LURJTMIESA-N	Endogenous metabolite	C18 pos.	180.10191	21.41000	M+H	95.54
Theophylline	2153	ZFXYFBGIUFBOJW-UHFFFAOYSA-N	Endogenous metabolite	C18 pos.	181.07201	36.93708	M+H	95.54
Paraxanthine	4687	QUNWUDVFRNGTCO-UHFFFAOYSA-N	Endogenous metabolite	C18 pos.	181.07201	35.62917	M+H	95.54
10-Hydroxydecanoate	74300	YJCJVMMDTBEITC-UHFFFAOYSA-N	Endogenous metabolite	C18 pos.	189.14853	222.42865	M+H	96.97
Indole-3-Methyl Acetate	74706	KTHADMDGDNYQRX-UHFFFAOYSA-N	Endogenous metabolite	C18 pos.	190.08626	244.97900	M+H	99.52

2-Hydroxypyridine	8871	UBQKCCHYAOITMY-UHFFFAOYSA-N	Endogenous metabolite	C18 pos.	191.08150	26.95000	2M+H	77.39
Caffeine	2519	RYYVLZVUVIJVGH-UHFFFAOYSA-N	Endogenous metabolite	C18 pos.	195.08766	52.82079	M+H	89.97
L-Dopa	6047	WTDRDQBEARUVNC-LURJTMIESA-N	Endogenous metabolite	C18 pos.	198.07609	24.20595	M+H	99.84
Myoinositol	892	CDAISMWEOUEBRE-UHFFFAOYSA-N	Endogenous metabolite	C18 pos.	203.05261	21.13000	M+Na	100.00
N,N-Dimethylarginine	123831	YDGMGEXADBMOMJ-LURJTMIESA-N	Endogenous metabolite	C18 pos.	203.15030	19.38000	M+H	99.84
O-Acetylcarnitine	439756	RDHQFKQIGNGIED-MRVPVSSYSA-O	Endogenous metabolite	C18 pos.	204.12304	23.00132	M+H	100.00
Mannitol	6251	FBPFZTCFMRRESA-KVTDHHQDSA-N	Endogenous metabolite	C18 pos.	205.06826	21.41000	M+Na	69.43
Sorbitol	5780	FBPFZTCFMRRESA-JGWLITMVSA-N	Endogenous metabolite	C18 pos.	205.06826	23.10313	M+Na	69.43
Xanthurenic acid; Xanthurenic acid	5699	FBZONXHGGPHHIY-UHFFFAOYSA-N	Endogenous metabolite	C18 pos.	206.04479	40.54000	M+H	30.10
N-Acetylserotonin	903	MVAWJSIDNICKHF-UHFFFAOYSA-N	Endogenous metabolite	C18 pos.	219.11281	62.86600	M+H	35.51
Pantothenic acid	6613	GHOKWGTUZJEAQD-ZETCQYMHSA-N	Endogenous metabolite	C18 pos.	220.11795	28.19000	M+H	99.04
N-Acetyltryptophan	2002	DZTHIGRZJZPRDV-LBPRGKRZSA-N	Endogenous metabolite	C18 pos.	247.10772	183.80569	M+H	98.57
5'-Deoxyadenosine	439182	XGYIMTFOTBMPFP-KQYNXXCUSA-N	Endogenous metabolite	C18 pos.	252.10912	25.93321	M+H	69.59
Deoxyadenosine	13730	OLXZPDWKRNYJJZ-RRKCRQDMSA-N	Endogenous metabolite	C18 pos.	252.10912	21.06000	M+H	69.59
Chenodeoxycholate	10133	RUDATBOHQWOJDD-BSWAIDMHSA-N	Endogenous metabolite	C18 pos.	273.18491	320.53868	M+H	98.41
Palmitate	985	IPCSVZSSVZVIGE-UHFFFAOYSA-N	Endogenous metabolite	C18 pos.	274.27405	234.91000	M+NH4	99.68
Glutarylcarnitine	71317118	NXJAXUYOQLTISD-SECBINFHSA-N	Endogenous metabolite	C18 pos.	276.14417	24.29128	M+H	99.20

Gamma-Linolenate	5280933	VZCCETWTMQHEPK-QNEBEIHSSA-N	Endogenous metabolite	C18 pos.	279.23186	322.85153	M+H	98.41
Hexadecanol	2682	BXWNKGSJHAJOGX-UHFFFAOYSA-N	Endogenous metabolite	C18 pos.	284.29480	404.37526	M+ACN+H	98.25
Retinoate	444795	SHGAZHPCJJPHSC-YCNIQYBTSA-N	Endogenous metabolite	C18 pos.	301.21621	353.03630	M+H	90.29
Sphinganine	91486	OTKJDMGTUTTYMP-ZWKOTPCHSA-N	Endogenous metabolite	C18 pos.	302.30536	234.91000	M+H	99.68
Stearate	5281	QIQXTHQIDYTFRH-UHFFFAOYSA-N	Endogenous metabolite	C18 pos.	302.30535	252.01945	M+NH4	99.68
Arachidate	10467	VKOBVWXKNCXXDE-UHFFFAOYSA-N	Endogenous metabolite	C18 pos.	330.33665	234.91000	M+NH4	100.00
3-Alpha,11-Beta,17,21-Tetrahydroxy-5-Beta-Pregnan-20-One	44725717	AODPIQQILQLWGS-OBRSLYEHSA-N	Endogenous metabolite	C18 pos.	349.23840	227.44145	M+H-H2O	97.61
Erucate	5281116	DPUOLQHDNGRHBS-KTKRTIGZSA-N	Endogenous metabolite	C18 pos.	356.35230	361.23781	M+NH4	100.00
Cortisone	222786	MFYSYFVPBJMHGN-ZPOLXVRWSA-N	Endogenous metabolite	C18 pos.	361.20095	224.07435	M+H	99.84
Cortisol	5754	JYGXADMDTFJGBT-VWUMJDOOSA-N	Endogenous metabolite	C18 pos.	363.21660	221.61000	M+H	99.52
Riboflavin	493570	AUNGANRZJHBGPY-SCRDCRAPSA-N	Endogenous metabolite	C18 pos.	377.14556	61.36448	M+H	58.92
Cholate	221493	BHQCQFFYRZLCQQ-OELDTZBJSA-N	Endogenous metabolite	C18 pos.	391.28487	254.36386	M+H-H2O	85.83
Palmitoylcarnitine	461	XOMRRQXKHMYMOC-OAQYLSRUSA-N	Endogenous metabolite	C18 pos.	400.34214	276.52582	M+H	100.00
Biliverdin	5280353	RCNSAJSGRJSBKK-NSQVQWHSSA-N	Endogenous metabolite	C18 pos.	583.25511	247.98000	M+H	99.20
Thyroxine	5819	XUIIKFGFIJCVMT-LBPRGKRZSA-N	Endogenous metabolite	C18 pos.	777.69396	230.50842	M+H	99.20
1-Aminocyclopropanecarboxylate	535	PAJPWUMXBYXFCZ-UHFFFAOYSA-N	Endogenous metabolite	HILIC neg.	100.04040	325.91959	M-H	68.26
N-Metyl-Alanine	5288725	GDFAOVXKHJXLEI-VKHMYHEASA-N	Endogenous metabolite	HILIC neg.	102.05605	328.00204	M-H	99.04

2-Aminoisobutyrate	6119	FUOOLUPWFVMBKG-UHFFFAOYSA-N	Endogenous metabolite	HILIC neg.	102.05605	326.49349	M-H	99.04
Benzyl Alcohol	244	WVDDGKGOMKODPV-UHFFFAOYSA-N	Endogenous metabolite	HILIC neg.	107.05020	55.40000	M-H	98.72
Fumarate; Fumaric acid	444972	VZCYOOQTPOCHFL-OWOJBTEDSA-N	Endogenous metabolite	HILIC neg.	115.00368	327.17785	M-H	99.84
Guanidinoacetate	763	BPMFZUMJYQTVII-UHFFFAOYSA-N	Endogenous metabolite	HILIC neg.	116.04655	352.43236	M-H	100.00
Mesoxalate	10132	XEEVLJKYYUVTRC-UHFFFAOYSA-N	Endogenous metabolite	HILIC neg.	116.98294	342.29576	M-H	95.37
Allothreonine	99289	AYFVYJQAPQTCCC-HRFVKAFMSA-N	Endogenous metabolite	HILIC neg.	118.05096	342.15517	M-H	100.00
3-Hydroxybenzaldehyde	101	IAVREABSGIHHMO-UHFFFAOYSA-N	Endogenous metabolite	HILIC neg.	121.02950	64.03159	M-H	97.45
Heptanoate	8094	MNWFXJYAOYHMED-UHFFFAOYSA-N	Endogenous metabolite	HILIC neg.	129.09210	66.70000	M-H	100.00
Salicylamide	5147	SKZKKFZAGNVIMN-UHFFFAOYSA-N	Endogenous metabolite	HILIC neg.	136.04040	57.40000	M-H	98.72
4-Hydroxybenzoate	135	FJKROLUGYXJWQN-UHFFFAOYSA-N	Endogenous metabolite	HILIC neg.	137.02441	167.64688	M-H	84.05
3-Hydroxybenzoate	7420	IJFXRHURBJZNAO-UHFFFAOYSA-N	Endogenous metabolite	HILIC neg.	137.02440	50.40000	M-H	51.36
Salicylate	338	YGSDEFSMJLZEOE-UHFFFAOYSA-N	Endogenous metabolite	HILIC neg.	137.02440	51.40000	M-H	51.36
6-Hydroxynicotinate	72924	BLHCMGRVFXRYRN-UHFFFAOYSA-N	Endogenous metabolite	HILIC neg.	138.01966	293.00546	M-H	99.36
Caprylate	379	WWZKQHOCKIZLMA-UHFFFAOYSA-N	Endogenous metabolite	HILIC neg.	143.10780	65.90000	M-H	100.00
N-Methylaspartate	22880	HOKKHZGPKSLGJE-GSVOUGTGSA-N	Endogenous metabolite	HILIC neg.	146.04588	327.80000	M-H	99.04
Ribose	5779	HMFHBZSHGGEWLO-SOOFDHNKSA-N	Endogenous metabolite	HILIC neg.	149.04550	187.91708	M-H	86.44
Vanillin	1183	MWOOGOJBHIARFG-UHFFFAOYSA-N	Endogenous metabolite	HILIC neg.	151.04010	55.97907	M-H	97.13

Resorcinol Monoacetate	5055	ZZPKZRHERLGEKA-UHFFFAOYSA-N	Endogenous metabolite	HILIC neg.	151.04010	54.70000	M-H	97.13
3-Sulfinoalanine	1549098	ADVPTQAUNPRNPO-REOHCLBHSA-N	Endogenous metabolite	HILIC neg.	152.00230	333.11428	M-H	81.34
2,5-Dihydroxybenzoate	3469	WXTMDXOMEHJXQO-UHFFFAOYSA-N	Endogenous metabolite	HILIC neg.	153.01930	69.87628	M-H	99.84
Nonanoate	8158	FBUKVWPVBMHYJY-UHFFFAOYSA-N	Endogenous metabolite	HILIC neg.	157.12340	62.90000	M-H	95.85
3,4-Dihydroxyphenylacetate	547	CFFZDZCDUFSOFZ-UHFFFAOYSA-N	Endogenous metabolite	HILIC neg.	167.03498	251.36000	M-H	62.52
Homogentisate	780	IGMNYECMUMZDDF-UHFFFAOYSA-N	Endogenous metabolite	HILIC neg.	167.03500	67.30000	M-H	62.68
Diacetyl	650	QSJXEFYPDANLFS-UHFFFAOYSA-N	Endogenous metabolite	HILIC neg.	171.06630	57.04135	2M-H	99.68
Prenol	11173	ASUAYTHWZCLXAN-UHFFFAOYSA-N	Endogenous metabolite	HILIC neg.	171.13904	69.93079	2M-H	100.00
Decanoate	2969	GHVNFZFCNZKVNT-UHFFFAOYSA-N	Endogenous metabolite	HILIC neg.	171.13910	59.20000	M-H	100.00
D-Glucuronolactone	92283	UYUXSRADSPPKRZ-SKNVOMKLSA-N	Endogenous metabolite	HILIC neg.	175.02481	123.53911	M-H	84.85
Pyruvate	1060	LCTONWCANYUPML-UHFFFAOYSA-N	Endogenous metabolite	HILIC neg.	175.02480	173.29656	2M-H	46.89
Isobutyrate	6590	KQNPFQTWMSNSAP-UHFFFAOYSA-N	Endogenous metabolite	HILIC neg.	175.09758	79.74551	2M-H	100.00
L-Gulonolactone	439373	SXZYCXMUPBBULW-SKNVOMKLSA-N	Endogenous metabolite	HILIC neg.	177.04050	176.60000	M-H	100.00
Gluconolactone	7027	PHOQVHQSTUBQQK-SQOUGZDYSA-N	Endogenous metabolite	HILIC neg.	177.04046	158.89214	M-H	68.90
Tagatose	439312	LKDRXBCSQODPBY-OEXCPVAWSA-N	Endogenous metabolite	HILIC neg.	179.05610	265.80000	M-H	100.00
Methyl Vanillate	19844	BVWTXUYLKBHMOX-UHFFFAOYSA-N	Endogenous metabolite	HILIC neg.	181.05063	70.69364	M-H	99.84
4-Pyridoxate	6723	HXACOUQIXZGNBF-UHFFFAOYSA-N	Endogenous metabolite	HILIC neg.	182.04588	59.92946	M-H	100.00

4-Hydroxy-3-Methoxyphenylglycol	10805	FBWPWWWZWKPJFL-UHFFFAOYSA-N	Endogenous metabolite	HILIC neg.	183.06630	67.00000	M-H	96.01
Diethyl 2-Methyl-3-Oxosuccinate	97750	OQOCQBJWOCRPQY-UHFFFAOYSA-N	Endogenous metabolite	HILIC neg.	183.06570	65.90000	M-H2O-H	96.01
Quinic Acid	6508	MODKMHXGCGKTLE-UHFFFAOYSA-N	Endogenous metabolite	HILIC neg.	191.05611	318.30000	M-H	91.55
D-Pinitol	164619	DSCFFEYYQKSRSV-FEPQRWDDSA-N	Endogenous metabolite	HILIC neg.	193.07180	283.14409	M-H	82.93
Indole-3-Pyruvate	803	RSTKLPZEZYGQPY-UHFFFAOYSA-N	Endogenous metabolite	HILIC neg.	202.05096	79.65927	M-H	85.65
Succinate Semialdehyde	1112	UIUJIQZEACWQSV-UHFFFAOYSA-N	Endogenous metabolite	HILIC neg.	203.05610	340.69886	2M-H	100.00
Pentanoate	7991	NQPDZGIKBAWPEJ-UHFFFAOYSA-N	Endogenous metabolite	HILIC neg.	203.12888	74.99789	2M-H	97.93
Glycerate	439194	RBNPOMFGQQGHHO-UWTATZPHSA-N	Endogenous metabolite	HILIC neg.	211.04590	57.90000	2M-H	99.20
D-Ribose 5-Phosphate	439167	KTVPXOYAKDPRHY-SOOFDHNKSA-N	Endogenous metabolite	HILIC neg.	229.01187	354.17815	M-H	37.96
Cystine	67678	LEVWYRKDKASIDU-IMJSIDKUSA-N	Endogenous metabolite	HILIC neg.	239.01657	366.40000	M-H	87.40
Anserine	112072	MYYIAHXIVFADCU-QMMMGPOBSA-N	Endogenous metabolite	HILIC neg.	239.11496	373.54747	M-H	48.01
Cytidine	6175	UHDGCWIWMRVCDJ-XVFCMESISA-N	Endogenous metabolite	HILIC neg.	242.07824	283.08491	M-H	74.80
Linoleate	5280450	OYHQOLUKZRVURQ-HZJYTTRNSA-N	Endogenous metabolite	HILIC neg.	279.23300	56.40000	M-H	99.68
Petroselinate	5281125	CNVZJPUDSLNTQU-SEYXRHQNSA-N	Endogenous metabolite	HILIC neg.	281.24860	55.30000	M-H	99.68
Glycerol-Myristate	79050	DCBSHORRWZKAKO-UHFFFAOYSA-N	Endogenous metabolite	HILIC neg.	301.23840	57.10000	M-H	100.00
Docosahexaenoate	445580	MBMBGCFOFBJSGT-KUBAVDMBSA-N	Endogenous metabolite	HILIC neg.	327.23300	55.70000	M-H	99.84
Trehalose	7427	HDTRYLNUVZCQOY-LIZSDCNHSA-N	Endogenous metabolite	HILIC neg.	341.10893	366.03391	M-H	94.74

Sucrose	5988	CZMRCDWAGMRECN-UGDNZRGBSA-N	Endogenous metabolite	HILIC neg.	341.10890	345.84703	M-H	94.74
Tricosanoate	17085	XEZVDURJDFGERA-UHFFFAOYSA-N	Endogenous metabolite	HILIC neg.	353.34250	54.20000	M-H	90.27
Nervonate	5281120	GWHCXVQVJPWHRF-KTKRTIGZSA-N	Endogenous metabolite	HILIC neg.	365.34250	53.80000	M-H	89.15
Pregnenolone Sulfate	105074	BTZNNJMUKZKXTH-CQSZACIVSA-N	Endogenous metabolite	HILIC neg.	395.18977	46.52251	M-H	99.84
Acetoin	179	ROWKJAVDOGWPAT-UHFFFAOYSA-N	Endogenous metabolite	HILIC neg.	87.04515	324.81832	M-H	79.74
5-Valerolactone	10953	OZJPLYNZGCXSJM-UHFFFAOYSA-N	Endogenous metabolite	HILIC pos	101.05970	69.30000	M+H	45.61
Benzylamine	7504	WGQKYBSKWIADBV-UHFFFAOYSA-N	Endogenous metabolite	HILIC pos	108.08080	251.30000	M+H	100.00
Hypotaurine	107812	VVIUBCNYACGLLV-UHFFFAOYSA-N	Endogenous metabolite	HILIC pos	110.02700	275.00000	M+H	99.84
Glycine	750	DHMQDGOQFOQNFH-UHFFFAOYSA-N	Endogenous metabolite	HILIC pos	117.06590	300.40000	M+ACN+H	100.00
Betaine	247	KWIUHFFTVRNATP-UHFFFAOYSA-N	Endogenous metabolite	HILIC pos	118.08630	260.60000	M+H	100.00
4-Hydroxybenzaldehyde	126	RGHHSNMVTDWUBI-UHFFFAOYSA-N	Endogenous metabolite	HILIC pos	123.04410	71.30000	M+H	71.29
1-Phenylethanol	7409	WAPNOHKVXSQRPX-UHFFFAOYSA-N	Endogenous metabolite	HILIC pos	123.08040	69.30000	M+H	90.43
Leucine	6106	ROHFNLRQFUQHCH-YFKPBYRVSA-N	Endogenous metabolite	HILIC pos	132.10190	245.50000	M+H	99.84
Homocysteine	778	FFFHZYDWPBMWHY-UHFFFAOYSA-N	Endogenous metabolite	HILIC pos	136.04270	267.90000	M+H	99.84
Estradiol-17Alpha	68570	VOXZDWNPVJITMN-SFFUCWETSA-N	Endogenous metabolite	HILIC pos	137.09610	68.20000	M+2H	100.00
4-Aminobenzoate	978	ALYNCZNDIQEVRV-UHFFFAOYSA-N	Endogenous metabolite	HILIC pos	138.05500	77.30000	M+H	81.66
Phenylacetaldehyde	998	DTUQWGWMVIHBKE-UHFFFAOYSA-N	Endogenous metabolite	HILIC pos	138.09130	223.30000	M+NH4	100.00

3-Hydroxyphenylacetate	12122	FVMDYYGIDFPZAX-UHFFFAOYSA-N	Endogenous metabolite	HILIC pos	153.05460	70.20000	M+H	96.17
3-Hydroxyanthranilate	86	WJXSWCUQABXPFS-UHFFFAOYSA-N	Endogenous metabolite	HILIC pos	154.04990	80.10000	M+H	97.45
P-Octopamine	440266	QHGUCRYDKWKLMG-QMMMGPOBSA-N	Endogenous metabolite	HILIC pos	154.08630	302.50000	M+H	91.71
Allantoin; Allantoin	204	POJWUDADGALRAB-UHFFFAOYSA-N	Endogenous metabolite	HILIC pos	159.05130	224.20000	M+H	99.84
Tryptamine	1150	APJYDQYYACXCRM-UHFFFAOYSA-N	Endogenous metabolite	HILIC pos	161.10730	247.80000	M+H	99.84
5-Hydroxylysine	3032849	YSMODUONRAFBET-UHNVWZDZSA-N	Endogenous metabolite	HILIC pos	163.10770	391.30000	M+H	95.69
3-Methylhistamine	69520	CPAGZVLINCPJEH-UHFFFAOYSA-N	Endogenous metabolite	HILIC pos	167.12910	336.30000	M+ACN+H	84.69
Serotonin	5202	QZAYGJVTTNCVMB-UHFFFAOYSA-N	Endogenous metabolite	HILIC pos	177.10220	295.30000	M+H	91.07
Glucosamine	439213	MSWZFWKMSRAUBD-IVMDWMLBSA-N	Endogenous metabolite	HILIC pos	180.08660	349.40000	M+H	96.81
D-Mannosamine	440049	MSWZFWKMSRAUBD-CBPJZXOFSA-N	Endogenous metabolite	HILIC pos	180.08660	347.40000	M+H	96.81
Acetylcholine	187	OIPILFWXSMYKGL-UHFFFAOYSA-N	Endogenous metabolite	HILIC pos	185.08130	76.30000	M+K	94.26
Azelate	2266	BDJRBEYXGGNYIS-UHFFFAOYSA-N	Endogenous metabolite	HILIC pos	189.11210	70.20000	M+H	47.85
N,N,N-Trimethyllysine	440120	MXNRLFUSFKVQSK-QMMMGPOBSA-N	Endogenous metabolite	HILIC pos	189.15980	395.60000	M+H	99.68
Sorbose	439192	LKDRXBCSQODPBY-AMVSKUEXSA-N	Endogenous metabolite	HILIC pos	198.09720	77.40000	M+NH4	100.00
Ethyl 3-Ureidopropionate	3613685	BYCFJYNUBNQNIY-UHFFFAOYSA-N	Endogenous metabolite	HILIC pos	202.11860	389.30000	M+ACN+H	99.04
Lipoamide	863	FCCDDURTIIUXBY-UHFFFAOYSA-N	Endogenous metabolite	HILIC pos	206.06680	71.60000	M+H	96.65
Methyl Jasmonate	5281929	GEWDNTWNSAZUDX-WQMVXFAESA-N	Endogenous metabolite	HILIC pos	225.14850	69.00000	M+H	96.33

Carnosine	439224	CQOVPNPJLQNMDC-ZETCQYMHSA-N	Endogenous metabolite	HILIC pos	227.11390	403.30000	M+H	60.93
Deoxycytidine	13711	CKTSBUTUHBMZGZ-SHYZEUOFSA-N	Endogenous metabolite	HILIC pos	228.09790	301.80000	M+H	97.29
Picolinate	1018	SIOXPEMLGUPBBT-UHFFFAOYSA-N	Endogenous metabolite	HILIC pos	247.07130	237.70000	2M+H	100.00
4-Methylcatechol	9958	ZBCATMYQYDCTIZ-UHFFFAOYSA-N	Endogenous metabolite	HILIC pos	249.11210	67.30000	2M+H	41.95
Retinyl Palmitate	5280531	VYGQUTWHTHXGQB-FFHKNEKCSA-N	Endogenous metabolite	HILIC pos	263.23690	66.90000	M+2H	99.84
Deoxyguanosine	187790	YKBGVTZYEHREMT-KVQBGUIXSA-N	Endogenous metabolite	HILIC pos	268.10400	211.40000	M+H	44.50
Adenosine	60961	OIRDTQYFTABQOQ-KQYNXXCUSA-N	Endogenous metabolite	HILIC pos	268.10400	236.40000	M+H	44.50
Guanosine	6802	NYHBQMYGNKIUIF-UUOKFMHZSA-N	Endogenous metabolite	HILIC pos	284.09890	228.50000	M+H	70.33
Glutathione Reduced	124886	RWSXRVCMGQZWBV-WDSKDSINSA-N	Endogenous metabolite	HILIC pos	308.09110	237.30000	M+H	54.07
Raffinose	439242	MUPFEKGTMRGPLJ-ZQSKZDJDSA-N	Endogenous metabolite	HILIC pos	522.20290	271.80000	M+NH4	40.99
[2-Ethyl,2-OH-4.0]-2-Ethyl-2-hydroxybutyric acid	77199	LXVSANCQXSSLPA-UHFFFAOYSA-N	Long chain fatty acid	C18 neg.	131.07140	19.60000	M-H	98.89
[2-OH-6.0]-2-Hydroxyhexanoic acid	99824	NYHNVHGFPZAZGA-UHFFFAOYSA-N	Long chain fatty acid	C18 neg.	131.07140	29.40000	M-H	98.89
[C8.1]-2-Octenoic acid	5282713	CWMPPVPFLSZGCY-SREVYHEPSA-N	Long chain fatty acid	C18 neg.	141.09210	161.40000	M-H	97.13
[3,3-diMe-5.0]-3,3-dimethylpentanedioate	20984	DUHQIGLHYXLKAE-UHFFFAOYSA-N	Long chain fatty acid	C18 neg.	159.06630	19.00000	M-H	86.46
[C8.0]-3-Hydroxyoctanoic acid	26613	NDPLAKGOSZHTPH-UHFFFAOYSA-N	Long chain fatty acid	C18 neg.	159.10270	53.90000	M-H	99.84
[C10.1]-Cis-2-Decenoic acid	5356596	WXBXVVIUZANZAU-HJWRWDBZSA-N	Long chain fatty acid	C18 neg.	169.12340	237.80000	M-H	99.84
[C10.1]-9-Decenoic acid	61743	KHAVLLBUVKBTBG-UHFFFAOYSA-N	Long chain fatty acid	C18 neg.	169.12340	226.40000	M-H	99.84

[C11.1]-Undecylenic acid	5634	FRPZMMHWLSIFAZ-UHFFFAOYSA-N	Long chain fatty acid	C18 neg.	183.13910	37.40000	M-H	81.69
[C10.1]-10-hydroxy-2-decenoic acid	5312738	QHBZHVUGQROELI-SOFGYWHQSA-N	Long chain fatty acid	C18 neg.	185.11830	55.10000	M-H	90.13
[C11.0]-Undecanoic acid	8180	ZDPHROOEEOARMN-UHFFFAOYSA-N	Long chain fatty acid	C18 neg.	185.15470	265.80000	M-H	100.00
[C10.0]-10-Hydroxydecanoic acid	26612	FYSSBMZUBSBFJL-UHFFFAOYSA-N	Long chain fatty acid	C18 neg.	187.13400	71.10000	M-H	98.41
[C12.1]-5-Dodecenoic acid	5312378	IJBFSOLHRKELLR-FPLPWBNLSA-N	Long chain fatty acid	C18 neg.	197.15470	263.30000	M-H	100.00
[2t-12.1-diacid]-Dodec-2-enedioic acid	5283028	MAZWDMBCPDUFDJ-VQHVLOKHSA-N	Long chain fatty acid	C18 neg.	227.12890	37.60000	M-H	100.00
[C18.1]-17-Octadecynoic acid	1449	DZIILFGADWDKMF-UHFFFAOYSA-N	Long chain fatty acid	C18 neg.	279.23300	334.30000	M-H	98.89
[C-22.5]-Docosapentaenoic acid	6441454	AVKOENOBFIYBSA-WMPRHZDHSA-N	Long chain fatty acid	C18 neg.	329.24860	347.60000	M-H	99.68
[C22.4]-Docosatetraenoic acid	5497181	TWSWSIQAPQLDBP-DOFZRALJSA-N	Long chain fatty acid	C18 neg.	331.26430	365.60000	M-H	99.84
[C22.3]-Docosatrienoic acid	5312557	WBBQTNCISCKUMU-PDBXOOCHSA-N	Long chain fatty acid	C18 neg.	333.27990	397.10000	M-H	99.84
[C22.2]-13,16-docosadienoic acid	5312554	HVGRZDASOHMCSK-HZJYTTRNSA-N	Long chain fatty acid	C18 neg.	335.29560	438.90000	M-H	99.84
[C22.0]-Docosanoic acid	8215	UKMSUNONTOPOIO-UHFFFAOYSA-N	Long chain fatty acid	C18 pos.	358.36795	288.85429	M+NH4	100.00
[2-Me,4e-5.1]-2-Methyl-4-pentenoic acid	549519	HVRZYSHVZOELOH-UHFFFAOYSA-N	Long chain fatty acid	HILIC neg.	113.06080	68.60000	M-H	99.68
[2-Me-7.0]-2-Methylheptanoic acid	14475	NKBWMBRPILTCRD-UHFFFAOYSA-N	Long chain fatty acid	HILIC neg.	143.10780	58.90000	M-H	100.00
[C8.0]-Dihydrolipoate	421	IZFHEQBZOYJLPK-UHFFFAOYSA-N	Long chain fatty acid	HILIC neg.	207.05190	68.00000	M-H	96.49
[C26.0]-Hexacosanoic acid	10469	XMHIUKTWLZUKEX-UHFFFAOYSA-N	Long chain fatty acid	HILIC neg.	395.38950	53.20000	M-H	44.18
[C13.0]-Tridecanoic acid	12530	SZHOJFHSIKHZHA-UHFFFAOYSA-N	Medium chain fatty acid	C18 neg.	213.18600	304.60000	M-H	100.00

[12-methyl-C15.0]-12-Methyltetradecanoic acid	21672	XKLJLHAPJBUBNL-UHFFFAOYSA-N	Medium chain fatty acid	C18 neg.	241.21730	339.10000	M-H	100.00
[C15.0]-Pentadecanoic acid	13849	WQEPLUUGTLDZJY-UHFFFAOYSA-N	Medium chain fatty acid	C18 neg.	241.21730	345.70000	M-H	100.00
[11-OH-16.0]-2-Hydroxyhexadecanoic acid	11065598	JGHSBPIZNUXPLA-OAHLLOKOSA-N	Medium chain fatty acid	C18 neg.	253.21680	336.90000	M-H2O-H	99.84
[14-Me-15.0]-Isopalmitic acid	36247	ZONJATNKKGGVSU-UHFFFAOYSA-N	Medium chain fatty acid	C18 neg.	255.23300	368.20000	M-H	100.00
[14-Me-16.0]-14-Methylpalmitic acid	22207	FXUKWLSZZHVEJD-UHFFFAOYSA-N	Medium chain fatty acid	C18 neg.	269.24860	382.90000	M-H	100.00
[15Me-16.0]-15-Methylhexadecanoic acid	164860	IIUXHTGBZYEGHI-UHFFFAOYSA-N	Medium chain fatty acid	C18 neg.	269.24860	382.90000	M-H	100.00
[C18.4]-Stearidonic acid	5312508	JIWBIWFOSCKQMA-LTKCOYKYSA-N	Medium chain fatty acid	C18 neg.	275.20170	303.70000	M-H	94.27
[C18.2]-11-Octadecen-9-ynoic acid	5312688	VENIIVIRETXKSV-BQYQJAHWSA-N	Medium chain fatty acid	C18 neg.	277.21730	325.00000	M-H	99.36
[C18.2]-9Z,11E-Octadecadienoic acid	5280644	JBYXPOFIGCOSSB-GOJKSUSPSA-N	Medium chain fatty acid	C18 neg.	279.23300	347.40000	M-H	98.89
[C18.1]-6-Octadecenoic acid	5282754	CNVZJPUDSLNTQU-OUKQBFOZSA-N	Medium chain fatty acid	C18 neg.	281.24860	373.80000	M-H	99.84
[16-Me-17.0]-Isostearic acid	21859	XDOFQFKRPWOURC-UHFFFAOYSA-N	Medium chain fatty acid	C18 neg.	283.26430	426.30000	M-H	100.00
[C19.1]-10Z-Nonadecenoic acid	5312513	BBOWBNGUEWHNQZ-KTKRTIGZSA-N	Medium chain fatty acid	C18 neg.	295.26430	397.20000	M-H	100.00
[C18.1]-12-hydroxy-9-cis-octadecenoic acid	5282942	WBHHMMIMDMUBKC-XFXZXTDPSA-N	Medium chain fatty acid	C18 neg.	297.24350	276.90000	M-H	99.52
[C20.4]-Arachidonic acid	11722594	HQPCSDADVLFHHO-LTKCOYKYSA-N	Medium chain fatty acid	C18 neg.	303.23300	336.40000	M-H	99.36
[C20.3]-5Z,8Z,11Z Eicosatrienoic acid	5312531	UNSRRHDPHVZAHH-YOILPLPUSA-N	Medium chain fatty acid	C18 neg.	305.24860	361.00000	M-H	99.84
[C20.1]-Eicosenoic acid<a0>	5282768	BITHHVVYSMSWAG-KTKRTIGZSA-N	Medium chain fatty acid	C18 neg.	309.27990	428.00000	M-H	99.84
[2-OH-14.0]-2-Hydroxymyristic acid	1563	JYZJYKOZGGEXSX-UHFFFAOYSA-N	Medium chain fatty acid	C18 pos.	262.23767	222.24275	M+NH4	99.52

[C20.0]-Phytanic acid	26840	RLCKHJSFHOZMDR-UHFFFAOYSA-N	Medium chain fatty acid	C18 pos.	330.33665	271.15616	M+NH4	100.00
[C19.0]-Nonadecanoic acid	12591	ISYWECDDZWTKFF-UHFFFAOYSA-N	Medium chain fatty acid	HILIC neg.	297.27990	55.90000	M-H	86.12
[10-Me-18.0]-10-Methyloctadecanoic<a0>acid	65037	BEOUGZFCUMNGOU-UHFFFAOYSA-N	Medium chain fatty acid	HILIC neg.	297.27990	55.80000	M-H	86.12
[2t4t-6.0-diacid]-trans,trans-Muconic acid	5356793	TXXHDPDFNKHHGW-ZPUQHVIOSA-N	Medium chain fatty acid	HILIC pos	285.06050	393.10000	2M+H	99.84
Alpha-ketoisovaleric acid	49	QHKABHOOEWYVLI-UHFFFAOYSA-N	Organic acid	C18 neg.	115.04010	20.00000	M-H	95.22
Glutaconic acid	5280498	XVOUMQNXTGKGMA-OWOJBTEDSA-N	Organic acid	C18 neg.	129.01930	18.70000	M-H	100.00
2,3-Dihydroxybutanedioic acid	875	FEWJPZIEWOKRBE-UHFFFAOYSA-N	Organic acid	C18 neg.	149.00920	18.10000	M-H	72.29
Succinylacetone	5312	WYEPBHZLDUPIOD-UHFFFAOYSA-N	Organic acid	C18 neg.	157.05060	19.20000	M-H	88.85
2-Methylbutyrylglycine	193872	HOACIBQKYRHBOW-UHFFFAOYSA-N	Organic acid	C18 neg.	158.08230	19.80000	M-H	86.46
Indole-3-carboxylic acid	69867	KMAKOBLIOCQGJP-UHFFFAOYSA-N	Organic acid	C18 neg.	160.04040	38.50000	M-H	97.77
Phenylpyruvic acid	997	BTNMPGBKDVTSJY-UHFFFAOYSA-N	Organic acid	C18 neg.	163.04010	34.80000	M-H	99.68
4-Pyridoxolactone	151228	HHPDVQLBYQFYFA-UHFFFAOYSA-N	Organic acid	C18 neg.	164.03530	24.50000	M-H	100.00
3-(4-hydroxyphenyl)propanoic acid	10394	NMHMNPHRMNGLLB-UHFFFAOYSA-N	Organic acid	C18 neg.	165.05570	20.00000	M-H	100.00
Gallic acid	370	LNTHITQWFMADLM-UHFFFAOYSA-N	Organic acid	C18 neg.	169.01420	19.30000	M-H	64.33
3-Hydroxysuberic acid	22328017	ARJZZFJXSNJKGR-UHFFFAOYSA-N	Organic acid	C18 neg.	189.07680	17.00000	M-H	89.65
Quinic acid	6508	AAWZDTNXLSGCEK-LNVDRNJUSA-N	Organic acid	C18 neg.	191.05610	18.80000	M-H	92.20
Cinnamoylglycine-trans	709625	YAADMLWHGMUGQL-VOTSOKGWSA-N	Organic acid	C18 neg.	204.06660	58.10000	M-H	99.84
Indolelactic acid	92904	XGILAAMKEQUXLS-UHFFFAOYSA-N	Organic acid	C18 neg.	204.06660	28.50000	M-H	99.84
3-Hydroxydecanedioic acid	3017884	OQYZCCKCJQWHIE-UHFFFAOYSA-N	Organic acid	C18 neg.	217.10810	18.00000	M-H	79.46
3-Methylglutaric acid	12284	XJMMNTGIMDZPMU-UHFFFAOYSA-N	Organic acid	C18 pos.	129.05521	42.38039	M+H-H2O	89.65
2-Oxobutyric acid	58	TYEYBOSBBBHJIV-UHFFFAOYSA-N	Organic acid	C18 pos.	144.06552	26.84000	M+ACN+H	98.89
Indole-3-aldehyde	10256	OLNJUISKUQQNIM-UHFFFAOYSA-N	Organic acid	C18 pos.	146.06004	192.32221	M+H	68.15
5-Hydroxypipecolic acid	151730	RKEYKDXXZCICFZ-UHFFFAOYSA-N	Organic acid	C18 pos.	146.08117	22.91236	M+H	99.20
2-Hydroxyphenylacetic acid	11970	CCVYRRGZDBSHFU-UHFFFAOYSA-N	Organic acid	C18 pos.	153.05462	109.92945	M+H	97.77

Indole-3-propionic acid	3744	GOLXRNDWAUTYKT-UHFFFAOYSA-N	Organic acid	C18 pos.	190.08625	224.80519	M+H	99.52
2-Isopropylmalic acid	5280523	BITYXLXUCSKTJS-ZETCQYMHSA-N	Organic acid	C18 pos.	199.05769	53.29116	M+Na	88.85
N-acetyl-tyrosine	68310	CAHKINHBCWCHCF-JTQLQIEISA-N	Organic acid	C18 pos.	224.09173	44.84671	M+H	46.66
Succinic acid	1110	KDYFGRWQOYBRFD-UHFFFAOYSA-N	Organic acid	HILIC neg.	117.01930	306.50000	M-H	99.68
Malic acid	222656	BJEPYKJPYRNKOW-REOHCLBHSA-N	Organic acid	HILIC neg.	133.01420	332.00000	M-H	99.04
3-Methylcrotonylglycine	169485	PFWQSHXPNKRLIV-UHFFFAOYSA-N	Organic acid	HILIC neg.	156.06660	200.00000	M-H	99.84
3-Methyladipic acid	6999745	SYEOWUNSTUDKGM-YFKPBYRVSA-N	Organic acid	HILIC neg.	159.06630	316.70000	M-H	98.72
2-Octenedioic acid	6079438	BNTPVRGYUHJFHN-HWKANZROSA-N	Organic acid	HILIC neg.	171.06630	312.90000	M-H	99.68
Aconitic acid	643757	GTZCVFVGUGFEME-IWQZZHSRSA-N	Organic acid	HILIC neg.	173.00920	335.00000	M-H	100.00
Citric acid	311	KRKNYBCHXYNGOX-UHFFFAOYSA-N	Organic acid	HILIC neg.	226.99640	175.03369	M+Cl	41.47
Oxalacetic acid	970	KHPXUQMNIQBQEV-UHFFFAOYSA-N	Organic acid	HILIC pos	265.01900	337.90000	2M+H	68.42
2-Hydroxyglutaric acid	439939	HWXBTNAVRSUOJR-VKHMYHEASA-N	Organic acid	HILIC pos	297.08160	64.70000	2M+H	99.52
3-Hydroxyglutarate	181976	ZQHYXNSQOIDNTL-UHFFFAOYSA-N	Organic acid	HILIC pos	297.08160	65.10000	2M+H	99.52
Prometon	4928	ISEUFVQQFVOBCY-UHFFFAOYSA-N	Pesticides	HILIC neg.	449.31060	62.10000	2M-H	98.56
Secbumeton	33443	ZJMZZNVGNSWOOM-UHFFFAOYSA-N	Pesticides	HILIC neg.	449.31070	63.10000	2M-H	98.56
Terbumeton	36584	BCQMBFHBDZVHKU-UHFFFAOYSA-N	Pesticides	HILIC neg.	449.31080	64.10000	2M-H	98.56
Cycluron	16554	DQZCVNGCTZLGAQ-UHFFFAOYSA-N	Pesticides	HILIC pos	199.18050	70.50000	M+H	96.81
Aminocarb	16247	IMIDOCRTMDIQIJ-UHFFFAOYSA-N	Pesticides	HILIC pos	209.12850	208.20000	M+H	45.45
Aldicarb-sulfone	9570093	YRRKLBAKDXSTNC-WEVVVXLNSA-N	Pesticides	HILIC pos	223.07470	72.20000	M+H	79.74
Butoxycarboxim	9571009	CTJBHIROCMPUKL-WEVVVXLNSA-N	Pesticides	HILIC pos	223.07470	72.20000	M+H	79.74
Acetamiprid	213021	WCXDHFDTOYPNIE-UHFFFAOYSA-N	Pesticides	HILIC pos	223.07450	72.10000	M+H	79.74
Monocrotophos	5371562	KRTSDMXIXPKRQR-AATRIKPKSA-N	Pesticides	HILIC pos	224.06820	75.10000	M+H	86.44
Forchlorfenuron	93379	GPXLRLUVLMHHIK-UHFFFAOYSA-N	Pesticides	HILIC pos	248.05850	70.50000	M+H	98.25
Spiroxamine.1; Spiroxamine.2	86160	PUYXTUJWRLOUCW-UHFFFAOYSA-N	Pesticides	HILIC pos	298.27410	192.50000	M+H	70.49
Mefenacet	91716	XIGAUIHYSDTJHW-UHFFFAOYSA-N	Pesticides	HILIC pos	299.08490	67.80000	M+H	58.85
Furalaxyl	42504	CIEXPHRYOLIQQD-UHFFFAOYSA-N	Pesticides	HILIC pos	302.13870	68.30000	M+H	69.70

[2-OH,2-Me-4.0]-2-Hydroxy-2-methylbutyric acid	95433	MBIQENSCDNJOIY-UHFFFAOYSA-N	Short chain fatty acid	C18 neg.	117.05570	19.60000	M-H	99.52
[2-OH,4-Me-5.0]-2-Hydroxy-4-methylpentanoic acid	83697	LVRFTAZAXQPQHI-YFKPBYRVSA-N	Short chain fatty acid	C18 neg.	131.07140	29.20000	M-H	98.89
Estrone	5870	DNXHEGUUPJUMQT-CBZIJGRNSA-N	Steroid Hormones	C18 pos.	271.16926	265.80000	M+H	34.39
Androstenedione	6128	AEMFNILZOJDQLW-QAGGRKNESA-N	Steroid Hormones	C18 pos.	287.20056	270.31010	M+H	57.80
Testosterone	6013	MUMGGOZAMZWBJJ-DYKIIFRCSA-N	Steroid Hormones	C18 pos.	289.21621	262.32238	M+H	55.10
7alpha-hydroxy-3-oxo-4-cholestenoic acid	91746147	SATGKQGFUDXGAX-SLTBQWEQSA-N	Sterol	C18 neg.	429.30100	272.30000	M-H	99.84
Allocholic acid	160636	BHQCQFFYRZLCQQ-PGHAKIONSA-N	Sterol	C18 pos.	391.28487	252.70531	M+H-H2O	85.83
Diosgenin	99474	WQLVFSAGQJTQCK-VKROHFNGSA-N	Sterol	HILIC pos	415.32070	66.80000	M+H	90.75
Octenoyl-L-carnitine	70679121	LOSHAHDSFZXVCT-LXKVQUBZSA-N	NA	C18 pos.	286.20128	206.85031	M+H	99.84

Table S2: results for regressing asthma on each of the identified exogenous compounds, a single compound at a time. 8y, 12y, 16y refer to the age stratified analysis while ‘All’ refers to the pooled analysis. The 95% confidence interval is a Wald confidence interval not adjusted for multiple testing. P-value of 0.00 means <0.01. Similarly, an FDR or p-value of 1 means >0.99. FDR is false discovery rate as controlled by Benjamini-Hochberg procedure, OR is odds ratio, p is p-value, PFAS is per- and polyfluoroalkyl substances.

		p				***FDR***				***OR***				***95% confidence interval***			
Class	Compound name	All	8y	12y	16y	*All*	*8y*	*12y*	*16y*	*All*	*8y*	*12y*	*16y*	*All*	*8y*	*12y*	*16y*
PFAS	Perfluorooctanoic acid	0.35	0.10	0.03	0.99	0.96	0.44	0.26	1.00	1.26	0.36	2.12	0.99	0.78 – 2.05	0.11 – 1.19	1.06 – 4.25	0.42 – 2.32
PFAS	Perfluoroheptanesulfonic acid	0.70	0.01	0.47	0.90	0.96	0.11	0.81	1.00	0.95	0.25	1.20	1.02	0.71 – 1.26	0.09 – 0.67	0.73 – 1.98	0.7 – 1.5
PFAS	Perfluorotetradecanoic acid	0.91	0.15	0.97	0.34	0.96	0.51	0.97	1.00	1.01	0.87	1.00	1.08	0.91 – 1.11	0.72 – 1.05	0.86 – 1.17	0.92 – 1.26
PFAS	Perfluorotridecanoic acid	0.41	0.29	0.75	0.30	0.96	0.64	0.91	1.00	0.88	0.80	1.08	0.73	0.65 – 1.19	0.53 – 1.2	0.69 – 1.69	0.41 – 1.31
PFAS	Perfluorodecanoic acid	0.82	0.54	0.92	0.44	0.96	0.77	0.97	1.00	0.96	1.39	1.03	0.83	0.69 – 1.33	0.49 – 3.89	0.61 – 1.72	0.52 – 1.33
PFAS	Perfluorohexanesulfonic acid	0.88	0.25	0.41	0.70	0.96	0.59	0.77	1.00	0.99	0.88	1.07	0.97	0.9 – 1.09	0.7 – 1.1	0.91 – 1.25	0.84 – 1.12
PFAS	Perfluoroheptanoic acid	0.34	0.64	0.13	0.98	0.96	0.77	0.59	1.00	1.08	0.87	1.18	1.00	0.93 – 1.25	0.49 – 1.54	0.95 – 1.47	0.8 – 1.24
PFAS	Perfluorononanoic acid	0.69	0.32	0.05	0.48	0.96	0.67	0.34	1.00	1.08	0.57	1.99	0.83	0.73 – 1.6	0.19 – 1.71	0.99 – 3.98	0.49 – 1.41
PFAS	Perfluoropropanoic acid	0.87	0.74	0.97	0.51	0.96	0.81	0.97	1.00	0.98	1.09	0.99	0.88	0.78 – 1.23	0.66 – 1.79	0.7 – 1.4	0.61 – 1.28
PFAS	Perfluorobutanesulfonic acid	0.07	0.51	0.01	1.00	0.93	0.77	0.10	1.00	1.09	1.07	1.23	1.00	0.99 – 1.2	0.87 – 1.31	1.05 – 1.43	0.86 – 1.16
PFAS	Perfluorooctanesulfonic acid	0.55	0.01	0.24	0.31	0.96	0.11	0.77	1.00	0.91	0.24	1.32	0.78	0.66 – 1.25	0.08 – 0.72	0.83 – 2.1	0.48 – 1.26
PFAS	Perfluoroundecanoic acid	0.85	0.02	0.95	0.33	0.96	0.18	0.97	1.00	0.98	0.39	1.01	1.21	0.75 – 1.27	0.18 – 0.87	0.67 – 1.53	0.82 – 1.78
PFAS	Perfluorooctanesulfonyl fluoride	0.73	0.01	0.14	0.44	0.96	0.11	0.59	1.00	0.94	0.22	1.47	0.83	0.68 – 1.32	0.07 – 0.7	0.88 – 2.46	0.51 – 1.34
PFAS	N-Methylperfluorooctanesulfonamidoacetic acid	0.71	0.13	0.40	0.83	0.96	0.48	0.77	1.00	0.88	0.26	1.59	0.88	0.44 – 1.75	0.05 – 1.44	0.54 – 4.67	0.28 – 2.8
PFAS	2-Aminohexafluoropropan-2-ol	0.96	0.68	0.62	0.54	0.96	0.77	0.91	1.00	0.99	0.77	1.21	0.76	0.58 – 1.67	0.23 – 2.65	0.57 – 2.59	0.32 – 1.84
Phenol	4-Hydroxybenzoic acid	0.45	0.69	0.66	0.70	0.96	0.77	0.91	1.00	0.95	0.94	0.95	0.96	0.83 – 1.09	0.7 – 1.27	0.75 – 1.2	0.79 – 1.17
Pesticides	Forchlorfenuron	0.94	0.45	0.60	0.30	0.96	0.76	0.91	1.00	0.98	1.62	1.27	0.69	0.59 – 1.63	0.46 – 5.79	0.52 – 3.1	0.34 – 1.39
Pesticides	DEET	0.84	0.61	0.94	0.58	0.96	0.77	0.97	1.00	1.15	0.45	1.09	1.73	0.3 – 4.39	0.02 – 9.5	0.11 – 10.76	0.25 – 12.09
Pesticides	Aldicarb-sulfone	0.40	0.10	0.30	0.17	0.96	0.44	0.77	1.00	1.21	0.46	1.43	1.67	0.78 – 1.88	0.18 – 1.17	0.72 – 2.85	0.81 – 3.43
Pesticides	3-Hydroxycarbofuran	0.83	0.65	0.44	0.96	0.96	0.77	0.79	1.00	0.98	1.12	0.89	1.01	0.82 – 1.17	0.69 – 1.83	0.66 – 1.19	0.78 – 1.3
Pesticides	Propoxur	0.15	0.08	0.71	0.65	0.96	0.44	0.91	1.00	1.07	1.17	1.03	1.04	0.98 – 1.17	0.98 – 1.38	0.89 – 1.19	0.89 – 1.21
Pesticides	Isoprocarb	0.53	0.60	0.35	0.69	0.96	0.77	0.77	1.00	0.98	1.06	0.94	0.98	0.9 – 1.05	0.84 – 1.34	0.83 – 1.07	0.88 – 1.09

Pesticides	Fenobucarb	0.83	0.25	0.37	0.79	0.96	0.59	0.77	1.00	0.99	1.22	0.92	0.98	0.87 – 1.12	0.87 – 1.69	0.76 – 1.11	0.81 – 1.18
Pesticides	Cycluron	0.80	0.46	0.52	0.76	0.96	0.76	0.86	1.00	1.04	0.78	1.15	1.07	0.79 – 1.36	0.4 – 1.52	0.75 – 1.78	0.69 – 1.65
Pesticides	Acetamiprid	0.43	0.16	0.72	0.81	0.96	0.51	0.91	1.00	1.08	1.39	1.06	0.97	0.9 – 1.29	0.88 – 2.18	0.75 – 1.5	0.76 – 1.23
Pesticides	Furalaxyl	0.39	0.97	0.33	0.67	0.96	0.97	0.77	1.00	1.04	1.00	1.07	1.03	0.95 – 1.14	0.75 – 1.32	0.93 – 1.23	0.89 – 1.2
Pesticides	Spiroxamine.1	0.36	0.44	0.25	0.07	0.96	0.76	0.77	1.00	0.80	0.68	1.66	0.54	0.5 – 1.28	0.26 – 1.78	0.7 – 3.96	0.28 – 1.05
Pesticides	Prochloraz	0.50	0.89	0.26	0.92	0.96	0.94	0.77	1.00	0.90	0.95	0.74	1.02	0.67 – 1.21	0.44 – 2.05	0.45 – 1.24	0.68 – 1.53
Pesticides	Propamocarb	0.88	0.08	0.36	0.99	0.96	0.44	0.77	1.00	0.97	2.89	0.74	0.99	0.62 – 1.51	0.9 – 9.32	0.39 – 1.41	0.49 – 2.02
Pesticides	2-Isopropyl-6-methyl-4-pyrimidinol	0.82	0.21	0.80	0.87	0.96	0.59	0.94	1.00	0.99	0.83	1.02	1.01	0.88 – 1.1	0.62 – 1.11	0.85 – 1.24	0.87 – 1.18
Phenol	4-tert-Octylphenol	0.06	0.93	0.00	0.96	0.93	0.95	0.10	1.00	1.79	0.94	4.55	1.03	0.97 – 3.3	0.26 – 3.36	1.63 – 12.67	0.39 – 2.71
Phenol	Pentachlorophenol	0.89	0.23	0.72	0.87	0.96	0.59	0.91	1.00	1.01	1.17	0.98	0.99	0.93 – 1.09	0.91 – 1.5	0.88 – 1.1	0.87 – 1.12
Phenol	Bisphenol S	0.91	0.64	0.65	0.87	0.96	0.77	0.91	1.00	1.00	1.07	0.97	1.01	0.92 – 1.08	0.81 – 1.41	0.86 – 1.1	0.9 – 1.14
Phthalate	Monoisononyl phthalate	0.94	0.65	0.01	0.59	0.96	0.77	0.10	1.00	0.97	0.43	1.67	0.87	0.48 – 1.96	0.01 – 13.88	1.16 – 2.41	0.53 – 1.44
Other	Monoethyl phosphate	0.41	0.00	0.09	0.51	0.96	0.05	0.50	1.00	0.80	0.10	1.89	0.69	0.48 – 1.35	0.03 – 0.4	0.91 – 3.96	0.23 – 2.09
Phthalate	Mono-2-heptyl phthalate	0.33	0.56	0.30	0.54	0.96	0.77	0.77	1.00	1.07	0.90	1.10	1.07	0.94 – 1.21	0.62 – 1.3	0.92 – 1.32	0.87 – 1.31
Phthalate	Monocyclohexyl phthalate	0.00	0.34	0.01	0.00	0.10	0.67	0.10	0.05	3.79	0.46	8.11	12.68	1.59 – 9.06	0.1 – 2.23	1.72 – 38.29	2.67 – 60.16
Phthalate	Monobutyl phthalate	0.90	0.45	0.81	0.86	0.96	0.76	0.94	1.00	0.98	0.77	1.05	1.04	0.74 – 1.31	0.39 – 1.51	0.68 – 1.63	0.65 – 1.67

1

Table S3a: results of the identified pesticides and pathways analysis. A cell is colored red if the pathway was perturbed at FDR < 0.1. The FDR adjusted p-value is displayed in those cells. 1 = Aldicarb-sulfone, 2 = 3-Hydroxycarbofuran, 3 = Propoxur, 4 = Isoprocarb, 5= Cycluron, 6 = Acetamiprid, 7 = Furalaxyl, 8 = Spiroxamine, 9 =Prochloraz, 10 =Propamocarb, 11= 2-Isopropyl-6-methyl-4-pyrimidinol. Compounds not associated with any pathway are not shown.

**Pathway**	**1**	**2**	**3**	**4**	**5**	**6**	**7**	**8**	**9**	**10**	**11**
Alanine and Aspartate Metabolism			0.0426								
Androgen and estrogen biosynthesis and metabolism								0.0332			
Arachidonic acid metabolism	2.88e-06	0.00759	0.00128		0.00969			5.76e-14		0.00192	
Arginine and Proline Metabolism			0.00338								
Aspartate and asparagine metabolism		0.0177	0.00673								
Beta-Alanine metabolism		0.0807	0.00673					0.0157			
Bile acid biosynthesis										0.0821	
Butanoate metabolism		0.0177	0.0757							0.0651	0.00989
C21-steroid hormone biosynthesis and metabolism										0.00192	
CoA Catabolism						0.0853					
De novo fatty acid biosynthesis								0.000401			
Drug metabolism - cytochrome P450						0.00353	0.0353				
Galactose metabolism		0.0618									
Glutamate metabolism	0.0927		0.00234								
Glutathione Metabolism	0.065	0.0807	0.0319	0.0716							
Glycerophospholipid metabolism		0.0177						0.0157			
Glycine, serine, alanine and threonine metabolism		0.0871	0.0319								
Glycosphingolipid metabolism										0.0651	
Glyoxylate and Dicarboxylate Metabolism			0.0757								
Histidine metabolism			0.00673								
Leukotriene metabolism	0.000884							1.08e-06		0.0821	
Linoleate metabolism	3.53e-06	0.0871	0.00673	0.0415				0.000783		0.0651	
Lysine metabolism	0.0928			0.0415							
Methionine and cysteine metabolism			0.0538								
Nitrogen metabolism			0.00989								
Prostaglandin formation from arachidonate	0.000884	0.0807	0.0748		0.00969			2.67e-06			
Prostaglandin formation from dihomo gama-linoleic acid			0.0998					0.00224			
Putative anti-Inflammatory metabolites formation from EPA			0.0396								
Sialic acid metabolism		0.0807									
Tryptophan metabolism						2.82e-08					
Tyrosine metabolism				0.0415		3.13e-08					
Urea cycle/amino group metabolism		0.0277	0.0527						0.0901		
Vitamin A (retinol) metabolism			0.00673					1.73e-06			
Vitamin B5 - CoA biosynthesis from pantothenate						0.0905					
Vitamin B6 (pyridoxine) metabolism						0.0019					

Table S3b: results of the identified PFAS compounds and pathways analysis. A cell is colored red if the pathway was perturbed at FDR < 0.1. The FDR adjusted p-value is displayed in those cells. 1 = Perfluoroheptanesulfonic acid, 2 = Perfluorooctanoic acid, 3 = Perfluorotetradecanoic acid, 4 = Perfluorodecanoic acid, 5 = Perfluoroheptanoic acid, 6 = Perfluorononanoic acid, 7 = Perfluoropropanoic acid, 8 = Perfluorobutanesulfonic acid, 9 = Perfluorooctanesulfonic acid, 10 = 2-Aminohexafluoropropan-2-ol, 11 = Perfluorooctanesulfonyl fluoride, 12 = N-Methylperfluorooctanesulfonamidoacetic acid. Compounds not associated with any pathway are not shown.

Pathway	1	2	3	4	5	6	7	8	9	10	11	12
Alanine and Aspartate Metabolism		0.0415						0.0469				
Aminosugars metabolism							0.0197					
Androgen and estrogen biosynthesis and metabolism												0.0474
Arachidonic acid metabolism	6.3e-06	0.0415			0.0631	0.00069	1.7e-07		0.00117		0.0179	0.00213
Arginine and Proline Metabolism				0.0955				0.0187				
Aspartate and asparagine metabolism								0.0397		0.0567		
Beta-Alanine metabolism	0.00983	0.0334				0.0111	0.0253	0.0397		0.0631		0.0574
Butanoate metabolism		0.0749	0.0961		0.0217	0.0892		0.0794	0.00117	0.0294	0.033	
C21-steroid hormone biosynthesis and metabolism												0.0393
C5-Branched dibasic acid metabolism					0.0338							
Carnitine shuttle												0.0159
Chondroitin sulfate degradation										0.0143		
De novo fatty acid biosynthesis				0.00108								
Fatty Acid Metabolism				0.0955								
Glutamate metabolism				0.0955	0.0338		0.0482	0.0887		0.0377		
Glutathione Metabolism				0.0514			0.0298					
Glycine, serine, alanine and threonine metabolism								0.0498				
Glycolysis and Gluconeogenesis										0.0787		
Glycosphingolipid biosynthesis - ganglioseries										0.0192		
Glycosphingolipid biosynthesis - globoseries										0.0143		
Heparan sulfate degradation										0.0143		
Histidine metabolism	0.0426	0.057					0.0298	0.00057		0.0291		
Keratan sulfate degradation										0.0197		
Leukotriene metabolism	0.000588					0.0111	0.00128		0.0146			0.0601
Linoleate metabolism	0.000144	0.0439	0.0187		0.0338	0.0111	5.27e-08	0.00057			0.0392	0.00311
Lysine metabolism				0.0514				0.0794				
Methionine and cysteine metabolism										0.0689		
N-Glycan Degradation										0.0224		
Nitrogen metabolism		0.0334					0.0478	0.0834				
Prostaglandin formation from arachidonate	0.000325					0.00284	4.73e-05					0.00506
Pyrimidine metabolism										0.0642		
Pyruvate Metabolism								0.0794				
Sialic acid metabolism										0.0642		
Starch and Sucrose Metabolism										0.0197		
Tyrosine metabolism										0.0197		
Urea cycle/amino group metabolism										0.0607		
Vitamin A (retinol) metabolism	0.0282					0.021	0.0375		0.052			0.00492

Table S3c: results of the identified phenol compounds and pathways analysis. A cell is colored red if the pathway was perturbed at FDR < 0.1. The FDR adjusted p-value is displayed in those cells. * = also a pesticide. Compounds not associated with any pathway are not shown.

Pathway	4-tert-Octylphenol	Pentachlorophenol *	4-Hydroxybenzoic acid
Arachidonic acid metabolism		0.0377	
Arginine and Proline Metabolism			0.0214
Beta-Alanine metabolism			0.0279
Butanoate metabolism			0.0279
Carnitine shuttle	0.0161		
Glutamate metabolism			0.0279
Glutathione Metabolism			0.0214
Glycine, serine, alanine and threonine metabolism			0.0986
Glyoxylate and Dicarboxylate Metabolism			0.0279
Leukotriene metabolism		0.0923	
Purine metabolism			0.0986
Urea cycle/amino group metabolism			0.0398
Vitamin B3 (nicotinate and nicotinamide) metabolism			0.0214

Table S3d: results of the identified phthalate compounds and pathways analysis. A cell is colored red if the pathway was perturbed at FDR < 0.1. The FDR adjusted p-value is displayed in those cells.

Pathway	Monobutyl phthalate	Monoisononyl phthalate	Monocyclohexyl phthalate	Mono-2-heptyl phthalate
Androgen and estrogen biosynthesis and metabolism			0.0363	0.0158
Arachidonic acid metabolism			8.41e-07	
Arginine and Proline Metabolism		0.067	0.0397	
Butanoate metabolism				0.0896
C21-steroid hormone biosynthesis and metabolism			8.41e-07	0.0896
Glutamate metabolism			0.0547	
Glycerophospholipid metabolism	0.0166			
Histidine metabolism		0.00388		
Leukotriene metabolism			6.64e-05	
Linoleate metabolism			0.000232	
Omega-3 fatty acid metabolism			0.0363	
Prostaglandin formation from arachidonate			0.000198	
Tyrosine metabolism				0.0809
Vitamin A (retinol) metabolism			0.0363	

Table S3e: results of identified exogenous compounds and pathways analysis (for chemical classes with only 1 member). A cell is colored red if the pathway was perturbed at FDR < 0.1. The FDR adjust ed p-value is displayed in those cells.

Pathway	Monoethyl phosphate
Alanine and Aspartate Metabolism	0.0787
Arachidonic acid metabolism	0.00142
Aspartate and asparagine metabolism	0.0787
Butanoate metabolism	0.0787
Glycosphingolipid metabolism	0.0719
Leukotriene metabolism	0.0787
Linoleate metabolism	0.0787
Prostaglandin formation from arachidonate	0.023
Vitamin A (retinol) metabolism	0.0787

Table S3f: pathways associated with the case status of the serum sample. FDR = false-discovery rate; p is the Fisher p-value output from Mummichog.

Pathway	Number of metabolites in pathway	Number of metabolites in pathway significant	Significant hits expected	p	FDR
Tyrosine metabolism	160	31	15.1	1.14e-05	0.000695
Lysine metabolism	52	10	5.34	0.00991	0.302
Butanoate metabolism	34	9	4.23	0.0156	0.318
TCA cycle	31	6	3.46	0.0402	0.387
Prostaglandin formation from arachidonate	78	16	4.23	0.0411	0.387
Fatty acid oxidation, peroxisome	28	2	0.846	0.0417	0.387

Figure S1: Venn diagram that shows how many people supplied serum samples at the follow-up of 8, 12, and 16 years old. Overlap of sets indicates that a subject provided a serum sample at both of those follow-ups.

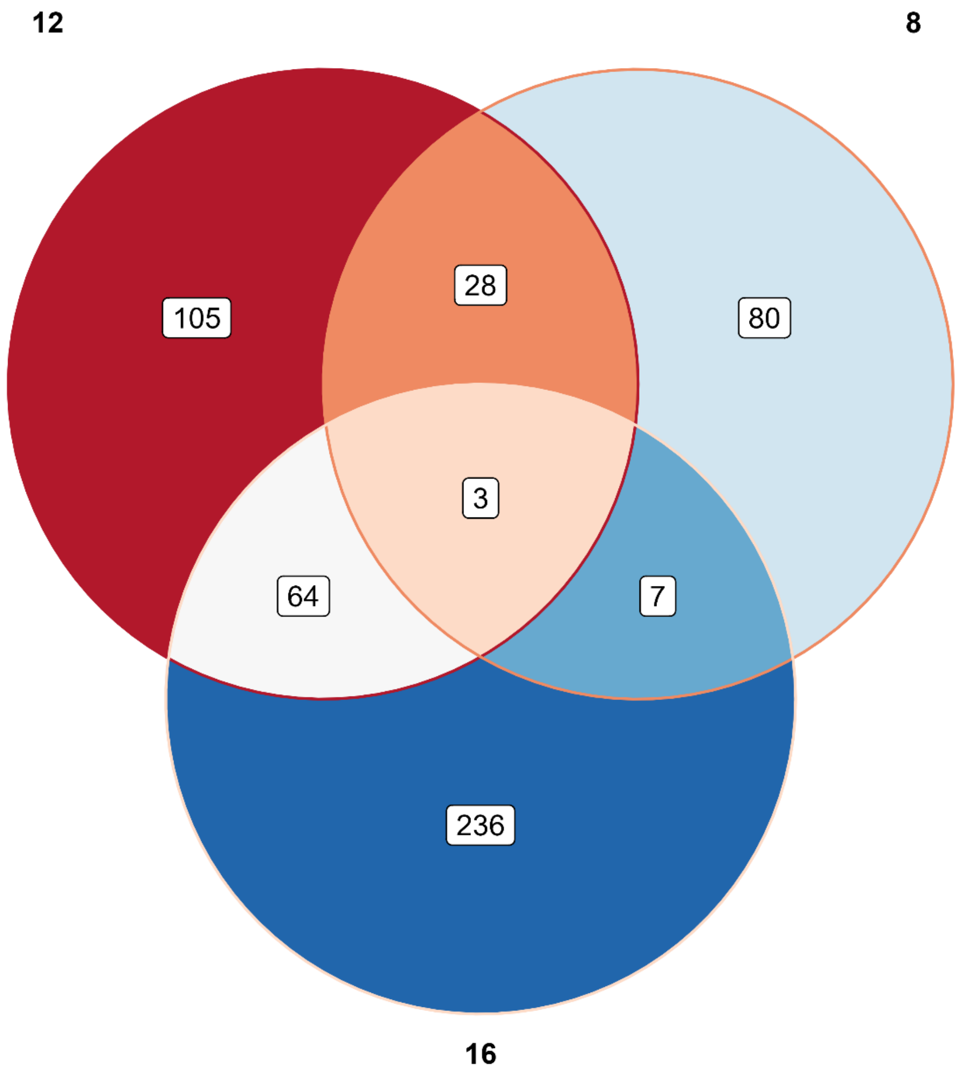

Figure S2: distribution of nondetects across all included 55,444 features.

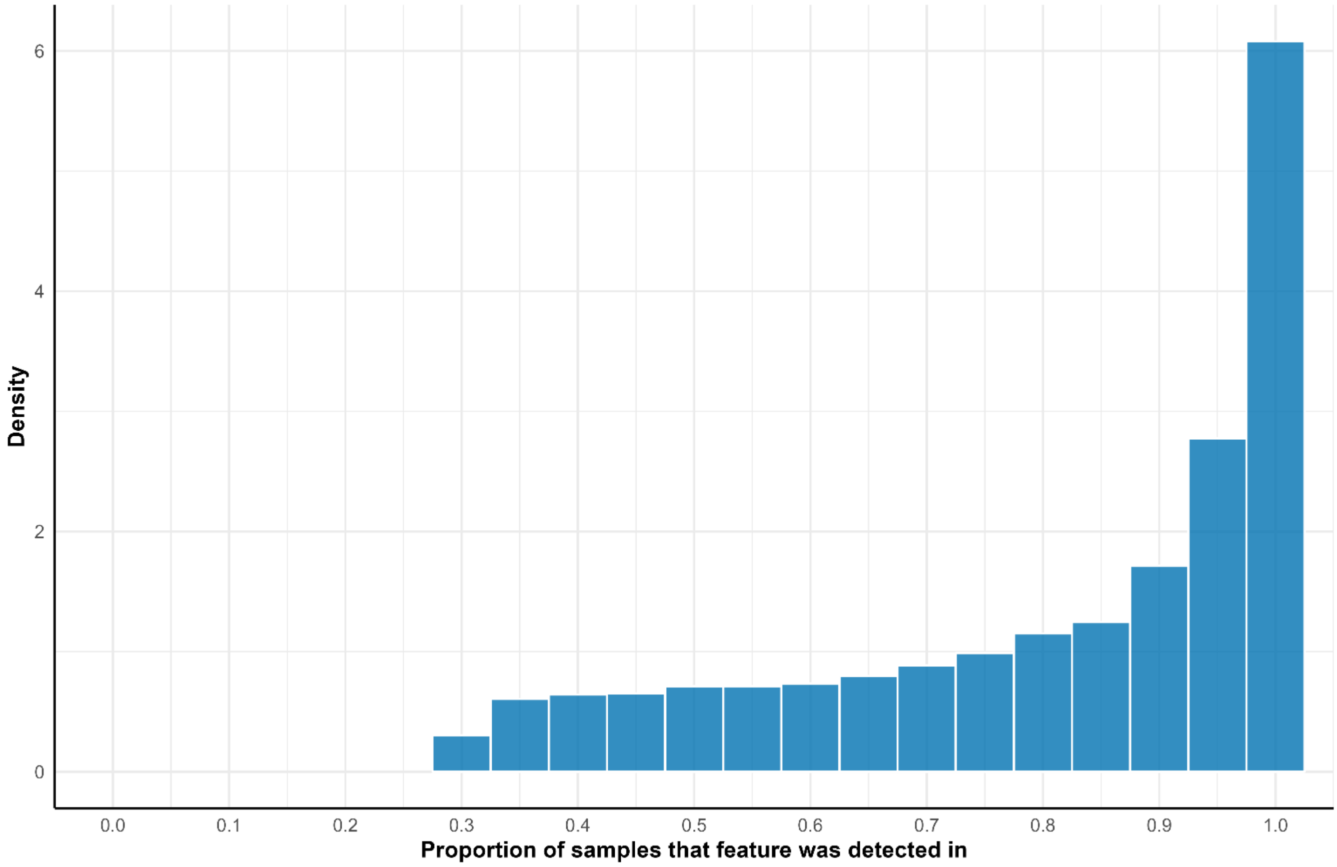

Figure S3: volcano plot of the association between case status and the identified compounds stratified by age of follow-up. (A) exogenous compounds (B) endogenous compounds. OR = odds ratio per one natural log unit change in compound intensity; FDR = false discovery rate as controlled by the Benjamini-Hochberg procedure; p = p-value; log is logarithm.

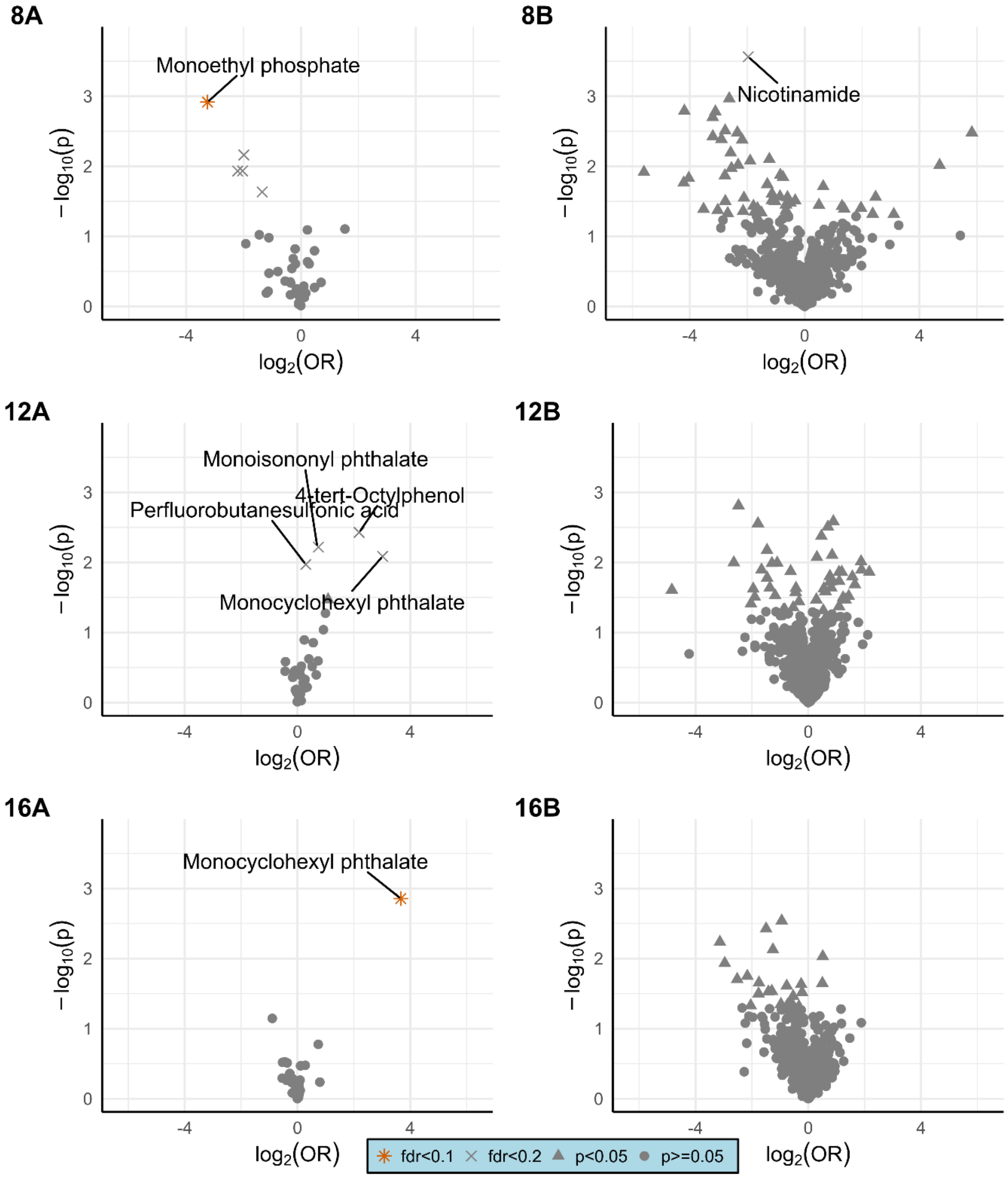

Figure S4: Spearman correlation coefficients of the identified exogenous compounds. PFAS = per-and polyfluoroalkyl substances.

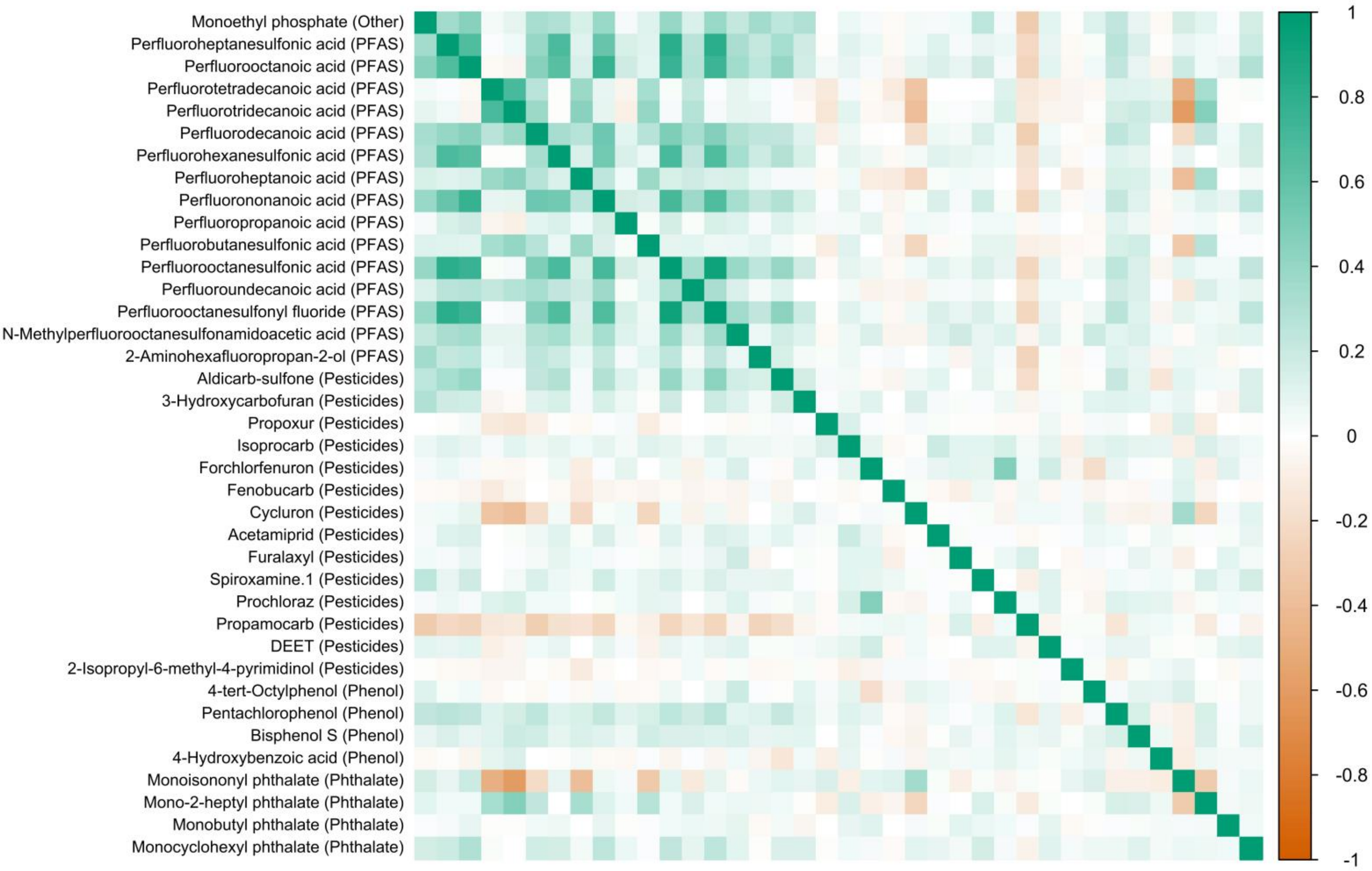

Figure S5: intraclass correlation coefficients (ICCs) over four years (age 12-16 years) for the identified exogenous compounds (more information on calculation in Supplementary Methodology III). The dotted line indicates the average ICC for the compounds in that chemical class. PFAS = per-and polyfluoroalkyl substances.

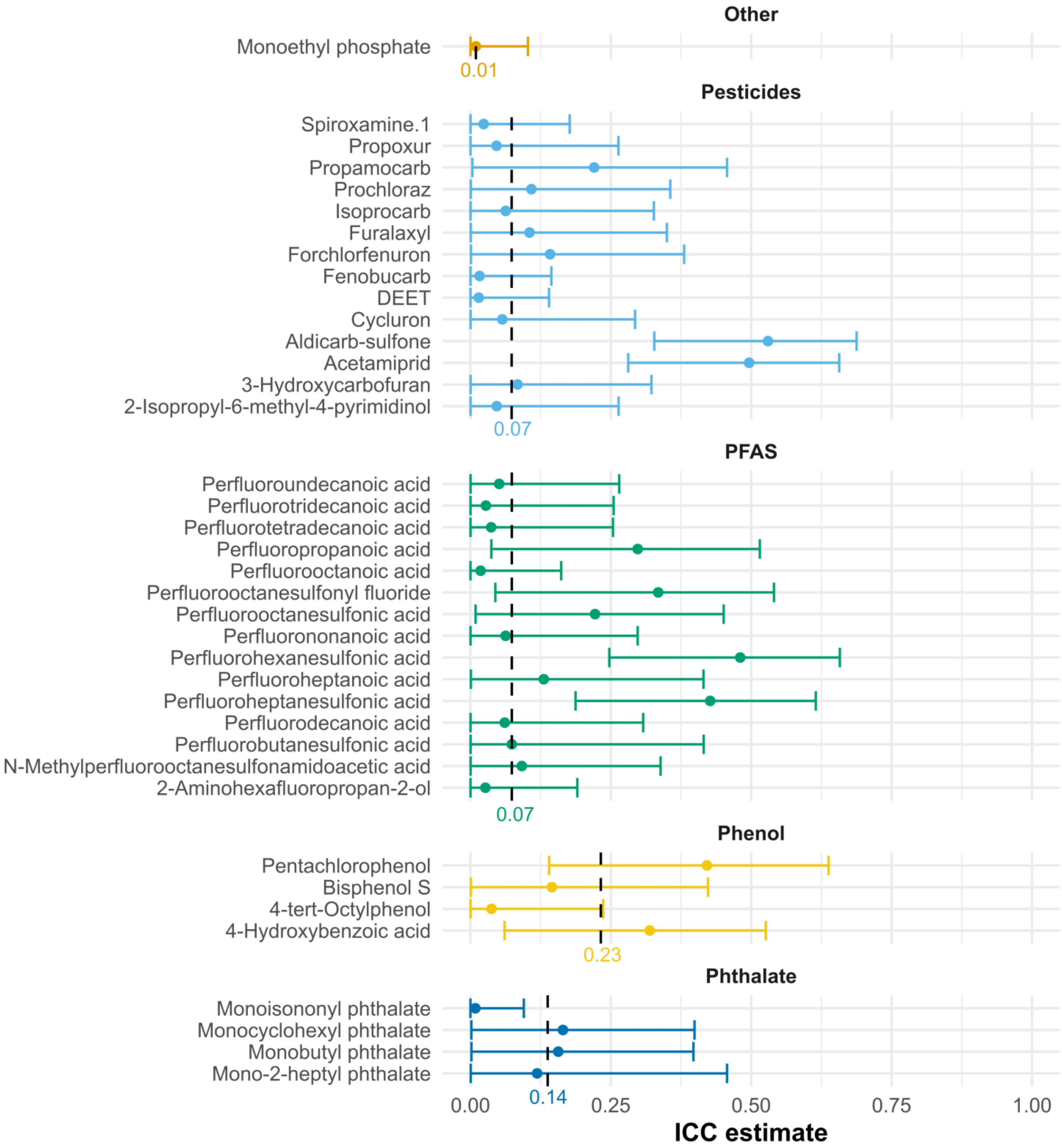

Supplement: Supplementary file 1 [file ee9-10-e480-s001.pdf]
